# Prostate cancer evolution from multilineage primary to single lineage metastases with implications for liquid biopsy

**DOI:** 10.1038/s41467-020-18843-5

**Published:** 2020-10-08

**Authors:** D. J. Woodcock, E. Riabchenko, S. Taavitsainen, M. Kankainen, G. Gundem, D. S. Brewer, P. Ellonen, M. Lepistö, Y. A. Golubeva, A. C. Warner, T. Tolonen, J. Jasu, W. B. Isaacs, M. R. Emmert-Buck, M. Nykter, T. Visakorpi, G. S. Bova, D. C. Wedge

**Affiliations:** 1 Big Data Institute, University of Oxford, Old Road Campus, Headington, Oxford, UK; 2 Faculty of Medicine and Health Technology, Tampere University and Tays Cancer Center, Tampere, FI 33014 Finland; 3 Medical and Clinical Genetics and Hematology Research Unit Helsinki, University of Helsinki and Helsinki University Hospital, Helsinki, Finland; 4 Department of Epidemiology and Biostatistics, Memorial Sloan Kettering Cancer Center, New York, NY USA; 5 Norwich Medical School, University of East Anglia, Norwich, UK; 6 Institute for Molecular Medicine Finland, University of Helsinki, Tukholmankatu 8, FIN-00290 Helsinki, Finland; 7 Cancer Genomic Research Laboratory (CGR), Division of Cancer Epidemiology and Genetics, NCI, FNLCR, Leidos Biomedical Research, Inc, Gaithersburg, MD USA; 8 Molecular Histopathology Laboratory, Frederick National Laboratory for Cancer Research, Frederick, MD 21702 USA; 9 Fimlab Laboratories, Department of Pathology, Tampere University Hospital, Tampere, Finland; 10 Brady Urological Institute, Johns Hopkins University School of Medicine, Baltimore, MD 21287 USA; 11 Laboratory of Pathology, National Cancer Institute, National Institutes of Health, Bethesda, MD USA; 12 Oxford NIHR Biomedical Research Centre, Oxford, UK; 13 Manchester Cancer Research Centre, University of Manchester, Manchester, UK; 14 Present Address: Avoneaux Medical Institute, Baltimore, MD USA

**Keywords:** Metastasis, Prostate cancer, Genome informatics, Molecular evolution, Cancer genomics

## Abstract

The evolutionary progression from primary to metastatic prostate cancer is largely uncharted, and the implications for liquid biopsy are unexplored. We infer detailed reconstructions of tumor phylogenies in ten prostate cancer patients with fatal disease, and investigate them in conjunction with histopathology and tumor DNA extracted from blood and cerebrospinal fluid. Substantial evolution occurs within the prostate, resulting in branching into multiple spatially intermixed lineages. One dominant lineage emerges that initiates and drives systemic metastasis, where polyclonal seeding between sites is common. Routes to metastasis differ between patients, and likely genetic drivers of metastasis distinguish the metastatic lineage from the lineage that remains confined to the prostate within each patient. Body fluids capture features of the dominant lineage, and subclonal expansions that occur in the metastatic phase are non-uniformly represented. Cerebrospinal fluid analysis reveals lineages not detected in blood-borne DNA, suggesting possible clinical utility.

## Abstract (web summary)

The evolutionary progression from primary to metastatic prostate cancer is largely uncharted, and the implications for liquid biopsy are unexplored. Here, the authors use deep genomic sequencing and histopathological information to trace tumor evolution both within the prostate and during metastasis in ten men.

## Introduction

Prostate cancer is the most commonly diagnosed cancer in men, and is responsible for ~10% of all cancer-related deaths in men^1,2^. The development of cancer within the prostate leads to a highly heterogeneous state with multiple disease foci^3^. These foci either correspond to tumors of independent origin^4^ or lineages resulting from evolutionary branches from a common ancestor^5^. Despite this intra-prostatic heterogeneity, evidence from ourselves^6^ and others^7,8^ indicates that metastases share a common ancestral or monoclonal origin in each patient.

The factors that contribute to metastatic potential remain unclear^9^. It has been reported that metastatic seeding can occur in waves from the prostate^10^ or through metastasis-to-metastasis seeding, often resulting in polyclonal seeding^11^. In either scenario, the metastasizing lineage continues to evolve after initial dissemination, leading to heterogeneity between metastases^10–12^. The evolutionary pressures affecting metastasising lineages are undetermined. However, metastases typically demonstrate convergent evolution of AR amplification following androgen deprivation therapy (ADT)^7,13^.

These complex patterns of heterogeneity confound our attempts to accurately predict the eventual metastatic state from primary samples, and thus determine prognosis and effective treatment strategies^14–16^. Indeed, it has been shown that genetic alterations seen in index lesions identified at biopsy do not consistently match those in metastases^16,17^. Similarly, liquid biopsies have been shown to provide information on clinically-relevant markers in metastatic disease^18^, but it remains unclear how representative they are of the evolutionary path and overall genomic status of the extant tumor cell population in a given patient^10,19^.

To date, most prostate cancer studies involving the direct reconstruction of evolutionary trajectories from multiple samples have been restricted to localized disease^5^, or concentrated on post-metastatic evolution using predominantly metastatic samples^10,11^, typically following ADT^20^. Combined investigations of both intra- and extra-prostatic evolution have been limited to single patients^10,13,16^. In this study we use deep genomic sequencing and histopathological information to trace tumor evolution both within the prostate and during metastasis in ten men. From this detailed tracing, we aim to reconcile two previous observations, that multiple metastases commonly have a single monoclonal origin and that considerable genetic heterogeneity is observed within and across both primary and metastases. We then explore the ramifications of our findings in circulating tumor DNA (ctDNA) derived from blood, as well as tumor DNA in cerebrospinal fluid (CSF) and urine.

## Results

### Reconstructing evolutionary origins of metastatic lineages

We performed deep (average 785X) targeted sequencing of 51 fresh-frozen samples from 10 men, previously whole-genome sequenced (WGS)^11^, in addition to samples from multiple microdissected regions in formalin or alcohol-fixed samples taken from the prostate (*n* = 33), lymph nodes (*n* = 3), seminal vesicles (*n* = 1), bladder (*n* = 1), and frozen body fluids (*n* = 24) including serum, plasma, and CSF. Four of the patients had undergone radical prostatectomy prior to autopsy, five patients still had cancer in their prostate at time of death, and one displayed no detectable tumor in the prostate at time of death (A34). Samples were taken at various times throughout the treatment schedule of each patient, and at rapid autopsy (Supplementary Figs. 1–10, Methods and Supplementary Data 2).

**Fig. 1:**
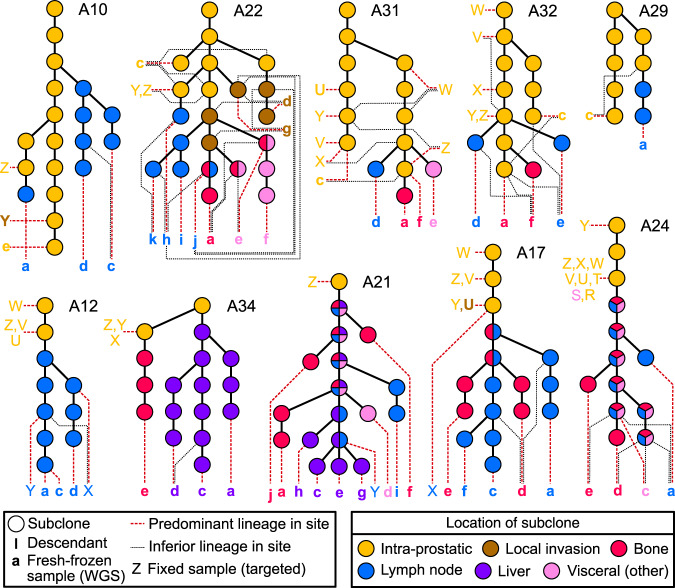
Phylogenetic trees of primary and metastatic prostate cancer evolution in ten men. Top row—patients who still had prostate in place at time of death; bottom row—patients who had undergone radical prostatectomy (A12, A17, A21, A24) or had no discernible cancer in the prostate at time of death (A34). Patient identifiers are to the right of the root node of each tree, which represents the most recent common ancestor (MRCA) of all tumor cells. Dotted lines connect the final subclone of a lineage with a letter denoting the sample or samples in which it was observed. Samples connected to multiple lines arising from different subclones indicates subclonal intermixture in the prostate and local organs, or polyclonal seeding in the periphery. Yellow filled subclones were observed in samples taken from the prostate. Other colors denote the location of sample in which the subclone was observed as indicated in the key, where subclones consisting of multiple colors indicate that the subclone was not found in the prostate, but was observed in the metastatic locations with corresponding colors. Fresh frozen samples are denoted by bold lower case letters in ascending order from a; microdissected fixed samples are standard upper case in descending order from Z. Letters are colored by sample tissue type. Samples taken from the prostate and local organs are arranged to the left or right of each tree. Samples taken from distant metastases are arranged horizontally at the bottom of each tree. Visceral (other) includes adrenal glands (A21, A22, A31), diaphragm (A24), and sigmoid colon (A24). Corresponding sample names, time of collection, cluster information and locations are given in Supplementary Figs. 1–10 and Supplementary Data 1 and 2.

Targeted regions covered SNVs and indels representative of each subclone previously identified from WGS^11^, as well as all coding mutations (“Methods” and Supplementary Data 4–6). The median number of mutations per sample used in this study was 705.5, with range 292–2032. We inferred subclonal structure from all tissue samples using the DPclust method^21^ (Supplementary Figs. 1–10), and constructed phylogenetic trees for all ten men (Fig. 1) following the Sum and Crossing Rules^22^ (Methods). As the targets were drawn from mutations identified in the samples sequenced previously, it was not possible to infer lineages absent from those samples. However, the sequencing of additional samples and the increased coverage enabled the resolution of many more distinct subclones in both primary and metastatic lineages.

### Tumor evolution occurs principally within the prostate

Many genomic alterations that characterize prostate cancer have been identified previously^7,23^. These events were identified in many of our samples, predominantly occurring prior to metastasis^11^. In all cases where multiple primary samples were studied, we identified branching into multiple lineages within the prostate, either through phylogenetic tree reconstruction from SNVs (Fig. 1), or through the identification of distinct copy number alterations (CNAs) determined from off-target reads^24^ (Supplementary Figs. 11 and 12). After metastatic dissemination had occurred, there were few additional driver events aside from the previously reported AR amplifications^11^ and mutations^13^.

Recently, mutational signatures^25^ have been used to estimate chronological timing of evolutionary events^26^. We adapted this technique to estimate the proportion of time spent in localized and metastatic phases of the disease (Methods). With the exception of A10, this indicated a relatively long period of monoclonal evolution in the prostate, followed by branching and then relatively quickly by subsequent metastatic dissemination (subfigure e, Supplementary Figs. 1–10). In samples where we had multiple intra-prostatic and metastatic samples, we identified more distinct subclones within the prostate and local organs than in the metastases (yellow and brown subclones; top row; Fig. 1). Furthermore, in patients with multiple intra-prostatic lineages, each lineage underwent several subclonal expansions in the prostate prior to initial metastasis, indicated by the chains of yellow subclones in Fig. 1.

### Lineages can be intermixed in the prostate and local organs

**Fig. 2:**
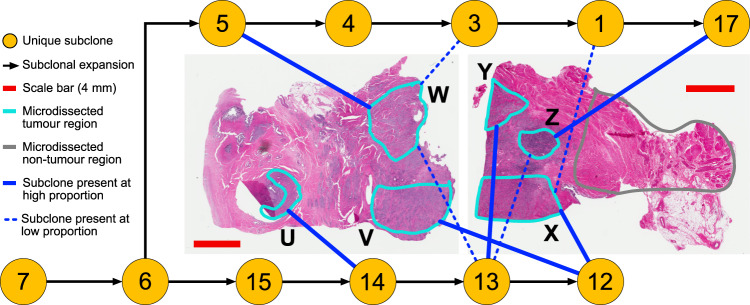
Subclones from distinct lineages can be spatially intermixed within the prostate. Six samples were collected across two tissue blocks from adjacent regions of the prostate. The intra-prostatic portion of the phylogenetic tree of A31 is shown, with lines denoting the final subclone of each distinct lineage found in the corresponding regions in each slide. Numbered subclones in yellow circles correspond to the clusters shown in Supplementary Fig. 8. Subclones that correspond to over 50% of tumor cells in a region are designated high proportion, all other subclones present are low proportion. Location of slides are given in Supplementary Fig. 13.

Cross-referencing the phylogenetic trees (Fig. 1) with prostatic location and histology demonstrated that subclones can exist in spatially distinct areas, but we also observed subclonal intermixture in A22, A29, A31, and A32, indicating that this is common. A31, from which we sampled six microdissected regions (Fig. 2, Supplementary Fig. 13), displayed the most frequent intermixture. We resolved two branched lineages, and three of the six tumor regions (Z, X, W) sampled contained cells belonging to both lineages (Fig. 2).

**Fig. 3:**
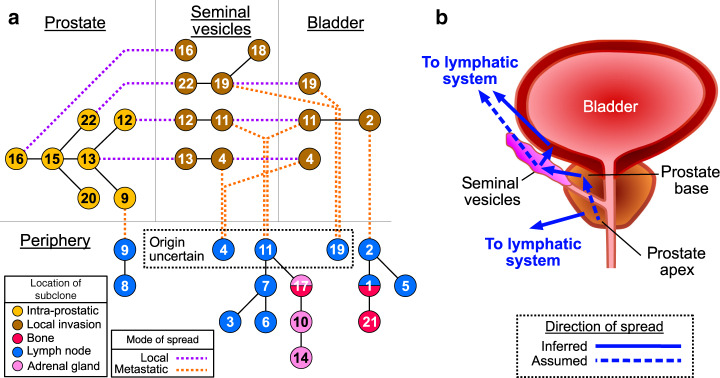
Multi-clonal invasion into adjacent organs and subsequent metastatic seeding in A22. Subclones identified in each sample were considered in conjunction with the phylogenetic trees to distinguish invasive local spread, evolutionary progression, and origins of metastatic dissemination. **a** A compound phylogenetic/compartmental diagram where lineages can move between compartments (dotted lines, purple = local invasion, orange = distant spread) and undergo subclonal expansion (solid black lines). Compartments were Prostate, Seminal Vesicles, Bladder and Periphery as labeled. Subclones are denoted by circles colored by tissue type, with numbers corresponding to clusters in Supplementary Fig. 5. **b** Spread of metastatic lineage occurred first through transprostatic migration to the base of the prostate, then to seminal vesicles and then to the bladder. At least one metastatic lineage (corresponding to subclone 2) was seeded from the bladder, and lineages identified through subclones 4, 11, and 19 may have originated from either the bladder or seminal vesicles (denoted ‘origin uncertain’). An additional metastatic lineage (subclone 9) appeared to have seeded directly from the prostate.

Surprisingly, subclonal intermixture was maintained during spread to local organs. In A22, cancer samples from the prostate apex, transrectal biopsy, seminal vesicles and the bladder contained subclones corresponding to different stages of common lineages. Several distinct lineages that originated in the prostate infiltrated the seminal vesicles, where the lineages identified by subclones 12, 13, and 22 underwent further evolution before invading the bladder (Fig. 3a). Each of these lineages spread to local lymph nodes, after which the lineage including subclone 11 spread to more distant sites. It is uncertain if all distant metastases from these lineages originated in the bladder, but the lack of lineages descended from subclones unique to the seminal vesicles in the metastases indicate this as the most likely scenario. These observations are consistent with concurrent trans-prostatic expansion of multiple lineages from the apex to the base and into the seminal vesicles and subsequently the bladder as a single tumoral mass (Fig. 3b). Similarly, in A10 three different subclones, albeit on the same lineage, were identified with differing cancer cell fractions (CCF) between the prostate and the bladder infiltrating metastasis (subclones 3, 4, and 6, Supplementary Fig. 1), suggesting simultaneous infiltration by multiple distinct cell subpopulations.

In A10, distant metastases arose from lineages found only in the primary tumor, and not from invasive spread to the bladder (sample Y, Fig. 1). Conversely, distant metastases in A22 appear to have originated from areas of local spread, although some lineages remained confined to the seminal vesicles (Fig. 3). Thus, even where tumor cells were likely present in the seminal vesicle and bladder for long periods of time prior to death, locally invasive cells did not always directly progress to distant metastases.

### Distant metastases derive from one intra-prostatic lineage

In four cases (A12, A17, A21, A29), the phylogenetic trees (Fig. 1) indicate that all metastases arose from one seeding event from the prostate or seminal vesicles. In a further three cases (A10, A31, A34) we observed two seeding events from one lineage, as evidenced by metastases derived from two distinct subclones of the same intra-prostatic branch (Fig. 1).

In A32, all distant metastases contained cells from one lineage (Fig. 1). In addition, metastatic sample **e** also contained cells from a second lineage, at low CCF (0.21). A22 branched into five lineages within the prostate and local organs (Figs. 1 and 3), and we traced at least four seeding events. Again, one lineage was present in all distant metastases (MRCA subclone 12, Supplementary Fig. 5a). Extensive metastasis-to-metastasis polyclonal seeding occurred within this lineage, but local pelvic lymph node samples **k** and **h** also displayed seeding from a separate lineage identified by subclone 9 (Supplementary Fig. 5a) at CCF 0.05 and 0.52 respectively. Sample **h** therefore contains the only major subclone (CCF > 0.5) that did not arise from the lineage found in all metastases in any of the 9 patients A10, A12, A17, A21, A22, A29, A31, A32, and A34. We therefore propose that a lineage found in all metastases of each patient is dominant, either because it seeded first or it outcompeted cells from other lineages.

A24 is the only case where we observed branching into two distinct lineages in the prostate that both seeded metastases separately. This is evidenced by CNAs identified in samples R and S that displayed vastly different copy number profiles to the fresh frozen metastatic samples (Supplementary Fig. 12). Sample R was a prostate biopsy sample, whereas sample S was a metastatic sample obtained from a sigmoid colon serosal metastasis removed during surgery for bowel obstruction 5 months prior to death (Supplementary Fig. 6b). This means that metastatic sample S arose from the same lineage as found in R, in contrast to the other metastases sampled. This patient received radiotherapy to the prostate bed region three years after prostatectomy (Supplementary Fig. 6b). As such, it appears that the lineage that seeded S was eradicated. Indeed, the metastatic samples in this patient showed enrichment for deletions and balanced inversions characteristic of radiation-induced DNA damage^27^, indicating they arose after radiotherapy treatment.

A similar situation arose in A34, in which we identified two metastatic seeding events from the prostate. One of these produced a bone metastasis (**e**) that required sacral nerve root decompression, which was performed eleven years prior to death. However, the phylogenetic tree shows the lineage leading to the liver metastases resected at autopsy branched from the main intra-prostatic lineage before the branch leading to the founding of the bone metastasis (Fig. 1, Supplementary Fig. 10a). This patient received extensive radiotherapy and anti-androgen therapy around the time of the sacral nerve root decompression that caused PSA levels to drop to ~0.1 ng/ml (Supplementary Fig. 10b). Subsequent platinum-based chemotherapy also caused PSA levels to drop in this patient, albeit temporarily (Supplementary Fig. 10b). Although radical prostatectomy was not performed on this patient, no cancer was identified in autopsy prostate tissue, indicating that this treatment successfully eliminated disease in the prostate and the lineage that led to metastasis (**e**). As such, it appears that the lineage that led to the liver metastases was present in an undetected state in the periphery for 11 years. This shows that early seeding can lead to occult disease that can remain dormant for several years before becoming active again.

The results of A24 and A34 indicate that two lineages developed metastatic potential separately, one of which was eliminated through treatment. It is therefore likely that the metastatic lineages in these patients rose to dominance at separate times.

### Precursors of metastasis differ between patients

We compared genomic aberrations in the dominant lineage with those in the lineage confined to the prostate (A10, A31), or was found at low CCF in a single metastasis (A32). We found multiple genetic alterations unique to the dominant lineage in each case.

In A31 a number of important events occurred prior to the branch between the metastatic and intra-prostatic lineages. These were loss of heterozygosity (LOH) events affecting 6q (covering *MAP3K7*), 8p (*NKX3-1*), 12p (*CDKN1B*), 13q (*RB1*), and 17p (*TP53*), as well as a focal HD in 10q (*PTEN*). In the metastatic lineage, a focal LOH event covering the *PPP2R5A* gene was followed by a whole-genome duplication (WGD), and subsequent single-copy losses of chromosomes 1, 6, 11, 14, 15, and 16. The intra-prostatic lineage contained alternative driver events: nonsynonymous SNVs affecting *TP53* and *MLL3*; LOH of 16q and focal LOH events affecting *FOXP1* and *PDE4B*; and amplification of *FOXA1*. As these lineages spatially coexisted in the prostate (Fig. 2), they likely had similar access to blood and lymphatic vessels.

**Fig. 4:**
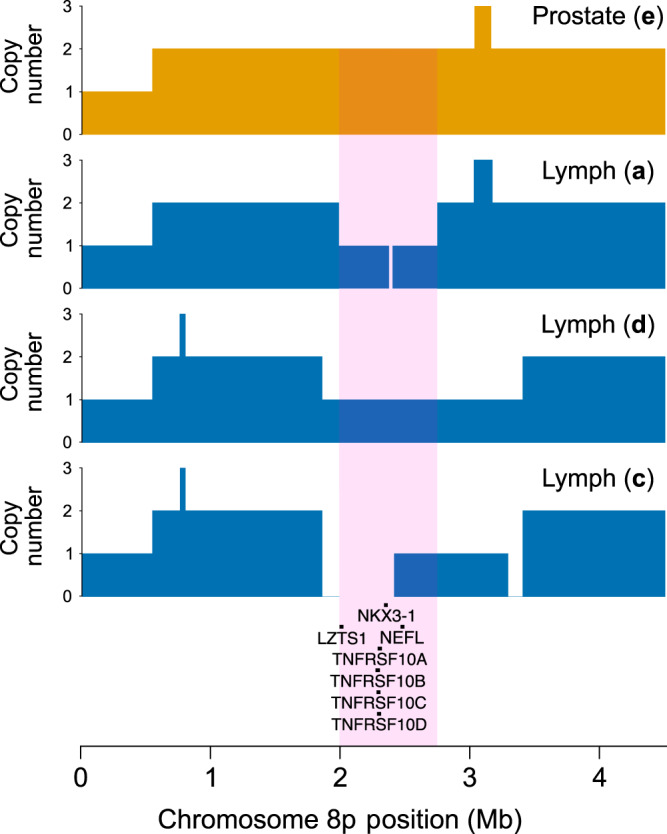
Copy number alterations covering the 8p21.3-8p21.2 locus in A10. Copy number profiles of the 8p region are shown by colored blocks, where the levels correspond to 1, 2, and 3 copies of the region, and no color indicates no copies are present. The region subject to loss of heterozygosity in all metastatic samples is highlighted in pink. Gene names of known drivers (see “Methods”) in this region are given below, with bars above detailing the corresponding genomic positions.

A10 underwent two branching events that resulted in formation of perigastric, periportal, and iliac lymph node metastases. The remaining intra-prostatic lineage continued to develop and underwent invasion into the bladder, but was not represented in any of the distant metastases. Analysing shared genomic alterations between the two metastatic lineages, we observed separate LOH events covering 8p21 (19,962,013–27,489,421; Fig. 4). This region contains a number of known driver genes including *NKX3-1*. Furthermore, from its absence in the Y and **e** samples, we determine that an 8p21 LOH never occurred in the main prostatic lineage. The presence of LOH covering exactly the same region (18,632,503–34,083,654) in **c** and **d** samples indicates that it occurred in a common progenitor of lineages in both samples (subclone 5; Supplementary Fig. 1a). The second branching event resulted in the lineage observed in transurethral resection sample Z, which subsequently populated the metastasis in the iliac lymph node, **a**. The LOH observed in **a** displayed different start and endpoints to the LOH common to **c** and **d** (Fig. 4), indicating that they arose from separate events. This CNA was not found in sample Z (Supplementary Fig. 14), meaning that both of the separate 8p LOH events in A10 were only observed in metastases.

**Fig. 5:**
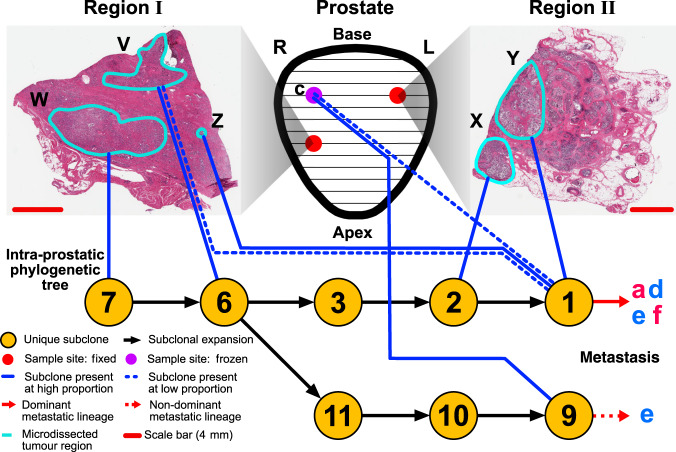
Metastasis-capable cells in multiple sites within the prostate and perineural regions in A32. The prostate (center) was divided into 13 slices (black lines) and three regions were sampled, two from slice 10, and one from slice 6. The purple circle denotes the region that was fresh frozen and sequenced as sample **c** and the red circles denote regions that were ethanol/methanol fixed and paraffin embedded. Histology slides shown to left and right, with microdissected regions highlighted in cyan with adjacent sequencing sample letters. The lower pane shows the phylogenetic tree of the intra-prostatic evolution, with numbers in the circles corresponding to subclonal clusters as derived in Supplementary Fig. 9. Lines joining the miscrodissected regions to a subclonal cluster indicate the final subclone observed in each region; these are designated as high (CCF > 0.5) and low (CCF < 0.5) proportion.

A32 also contained two branching lineages, one of which was the dominant lineage in all subsequent metastases, and the other was found in only one metastatic sample (**e**). Analysis of the histology of samples corresponding to the dominant lineage shows tumor cells growing in a perineural region in sample Z (Fig. 5); such perineural invasion (PNI) is associated with increased risk of metastasis as it provides a route out of the prostate^28^. Indeed, subclones growing around this nerve represent the most recent common ancestor of tumor cells found across all metastases. We investigated events that preceded metastasis in this lineage, and identified a missense mutation (S37C) in *CTNNB1* (from sample X, Y, and Z) in the metastatic lineage, despite relatively few other differences^11^.

### Evolution occurs linearly after metastasis

Evolution post-metastasis was consistent with multiple seeding events occurring from a single continually evolving lineage. This is most apparent in patient A21, where this lineage is defined by the subclones 15, 14, 11, 12, 10, 9, 16 (Supplementary Fig. 4a). All pairs of metastases have one of these subclones as a common ancestor. Similar behavior is also discernible in prostatectomy patients A17 and A24, as well as non-prostatectomy patients A31 and A32 (Fig. 1). Branches that did not share a single-lineage common ancestor were only observed in A10 (MRCA subclone 5; Supplementary Fig. 1a) and A22 (MRCA subclone 7; Supplementary Fig. 5). These results suggest that cells from a single dominant lineage continue to seed most, if not all, metastases after departing the prostate. The phylogenies of all patients are consistent with this hypothesis. However, it is not clear if seeding always occurs from a single site (i.e. the prostate in A31 and A32), or between metastatic sites in sequence^29^.

These patterns are indicative of linear evolution^30^, which is thought to occur when successive driver mutations confer a selective advantage that allows carriers to outcompete their ancestors^31^. In these metastases, there do not appear to be the selective sweeps that are associated with repeated waves of positive selection in this model. These results could therefore indicate negative selective pressure, or may be explained by particularly favorable access to the circulatory system by the dominant lineage.

### Metastasis-to-metastasis polyclonal seeding is common

Due to the inclusion of additional samples and the increased resolution afforded by our deep sequencing, we were able to identify more cases of polyclonal seeding between metastases than in our previous study^11^. Whereas polyclonal seeding was found in five of ten (A22, A24, A31, A32, A34) cases in the prior study, we found it occurred in an additional four patients (A10, A12, A17, A21); the only exception being A29 for which we only have one metastatic sample and so detection of metastasis-to-metastasis polyclonal seeding is not possible. This indicates that during metastasis-to-metastasis transmission polyclonal seeding is the norm. Polyclonal spread in the liver of A34 was detected. The individual samples were non-contiguous and physically separated by at least 1 cm, indicating that separate deposits arose from metastatic spread rather than local expansion.

### Variable representation of metastatic DNA in body fluids

**Fig. 6:**
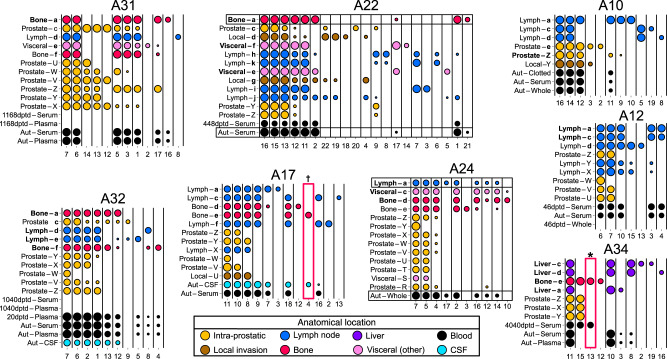
Subclones in tissue samples can be identified in DNA obtained from liquid biopsy. Plots showing the proportion of single nucleotide variants corresponding to each subclone (numbered on x-axis) that are observed in each sample (letters on y-axis). The area of the circle is proportional to the cancer cell fraction (CCF) of the subclone, with CCF of 1 found in the leftmost (truncal) nodes of the subplot. Anatomical location of tissue samples are also given in the sample label, and colored as in the legend. Body fluid samples are denoted by the time when the sample was taken (Aut = autopsy, dptd = the number of days prior to death), and the type of fluid (Serum; Plasma; blood, either Clotted or Whole; Cerebrospinal fluid (CSF)). Sample locations in bold are those that contain lineages represented in liquid biopsy at the highest CCF. Tissue samples in black boxes were polyclonal mixtures and contained similar subclonal CCFs to the boxed body fluid sample. Red boxes denote subclones of interest: star denotes subclones corresponding to a lineage found only in a spinal metastasis and serum, both of which were extracted 4040dptd, and dagger denotes a subclone that is only observed in a subdural metastasis and CSF.

To determine the information available in liquid biopsies, we examined six types of body fluid sample obtained at various times in the patients’ treatment cycles: CSF, whole and clotted blood samples, plasma, serum, and urine. Targeted DNA-sequencing of the body fluids of eight patients passed QC (Methods). We were able to identify tumor DNA in fluids derived from blood in all eight patients, as well as in the CSF of two of these. Urine of two patients was also sequenced, but we were not able to detect tumor DNA at sufficient levels for use in subsequent analyses. We cross-referenced DNA in the blood ctDNA and CSF with subclones previously identified in the tissue samples to investigate the representation of the samples in body fluids (Fig. 6; Supplementary Fig. 15).

When cancer subclones were detected in blood ctDNA, the truncal metastatic clone was always represented, and the subclones from different metastatic sites are variably represented. In blood taken at autopsy, in no case did the subclones in the blood match all known subclones identified in metastases from the same man. Subclones found only in prostate were never detected in blood. In 3/8 patients (A10, A12 and A31), we identified just one lineage in the ctDNA, whereas in 5/8 patients (A17, A21, A22, A32, A34), we were able to identify subclones from multiple lineages. Of these, blood samples taken at autopsy in A22 and A24 displayed subclonal proportions that were remarkably similar to a single tissue sample (black boxes, Fig. 6), indicating that this site or one with a similar composition may have seeded the tumor cells in the blood. One of these was situated in bone (A22) and the other in a lymph node (A24), so there is no indication that a particular tissue type is more amenable to hematological representation.

We sequenced CSF taken at autopsy in two patients: A17 and A32. In A32, CSF identifies 2/4 of the lineages that were found in the blood sample, with no additional lineages observed. In A17, we detected both lineages identified in the blood sample, and an additional lineage that corresponded to a metastasis in a subdural region of the cranium (red box †, Fig. 6). The observations of subclones present in CSF but not in blood, and vice versa, indicates ctDNA does not freely cross the blood-CSF barrier.

We had blood, serum or plasma samples at earlier time points in 5 patients. These were A12 – 46 days prior to death (dptd), A22 – 448dptd, A31 – 1186dptd, A32 – 1080dptd, A32 – 20dptd and A34 – 4040dptd. Of these, only the A31 – 1186dptd and A32 – 1080dptd samples did not display mutations consistent with the previously identified subclones. The subclones identified in the A12 – 46dptd and A32 – 20dptd were the same as those observed at autopsy, as was the A22 – 448dptd sample, although this was of poor quality. Remarkably, the A34 – 4040dptd sample contained subclones 15 and 13 that corresponded to the same lineage as the spinal metastasis that was removed 11 years prior to death (red box *, Fig. 6). These subclones were not observed in body fluid samples at autopsy, providing evidence that this lineage was eliminated by treatment (Supplementary Fig. 10b).

## Discussion

These findings provide numerous insights into prostate cancer evolution both intra-prostatically and during metastatic dissemination. It is clear that each individual cancer evolves in a unique way. However, there are some commonalities between these evolutionary trajectories. In particular, we consistently observe the development of multiple intra-prostatic lineages prior to the emergence of one dominant lineage that seeds many, if not all, of the subsequent metastases. This indicates that metastatic proliferation is relatively rare and likely subject to strong selective pressure.

In these advanced-stage cancers, extensive local disease was present. We found that divergent lineages spatially coexisted in the same regions of cancer within the prostate. These results contrast with those from studies of pancreatic^32^, renal^33^, glioblastoma^34^, and lung cancers^35^, where subclones were spatially distinct. We also found this subclonal intermixture was maintained during invasive spread to local organs. Remarkably, when spread to local organs was observed, these lineages did not necessarily lead to distant metastasis. Similar behavior has been observed in pancreatic cancer^32^, where metastatic colonization of the peritoneum and distant metastases are seeded by different lineages. If the locally invasive lineage is not always the metastatic lineage, stage T3 (extracapsular extension) may be an indirect marker for advanced disease, rather than a direct measure of the likelihood of metastatic dissemination.

We found that the majority of driver aberrations occurred in the trunk of the evolutionary tree. Branching into multiple lineages then occurred, after which one lineage initiated metastatic expansion. The metastatic and non-metastatic lineages therefore displayed relatively few differences, enabling the itemization of individual putative driver events. Lineages that did not metastasize continued to accumulate additional driver aberrations after diverging from the metastatic lineage. This was most notable in A31, where the non-metastatic lineage experienced six distinct known driver aberrations, compared to just 2 in the metastatic lineage. These results indicate that full metastatic potential does not arise through the cumulative onslaught of a large number of driver events, but rather the accumulation of a set of synergistic aberrations.

It is therefore possible that genetic factors, i.e., either or both of the heterozygous loss of *PPP2R5A* or the WGD, provided the crucial functional step toward metastatic potential in A31. This is further evidenced by the finding that WGD is significantly enriched in prostate cancer metastases compared to primary tumors^36^. Furthermore, we also found similar putative ‘metastatic trigger’ events in A10 and A32. Metastatic lineages in A10 displayed convergent evolution of LOH events affecting a small region of 8p covering the *NKX3-1* gene, which has been associated with aggressive disease^37^. This could indicate that this LOH was sufficient to initiate metastatic dissemination in this patient. In A32, we found the MRCA of cells in all metastatic lineages resided in a perineural region in the prostate; these had experienced a *CTNNB1* missense mutation. *CTNNB1* is a known driver gene that codes for the β-catenin protein, a component of the extra-cellular matrix^38^ and a constituent of the Wnt signaling pathway^39^. The precise mechanisms of perineural invasion remain unclear^40^, but it is well established that aberrant expression of Wnt/β-catenin targets *MMP2* and *MMP9* lead to an increased proclivity for perineural invasion in pancreatic cancer^41^. It is plausible that this genetic alteration facilitated spread to the perineural space, from where it seeded to distant sites. The accumulation of genetic alterations was very different across these three patients, indicating that there is no common route to metastasis. These results suggest that tumor evolution in the prostate leads to a primed state, after which the acquisition of a key genetic alteration could tip the balance toward metastasis. It is also possible that epigenomic or other unmeasured factors contributed to the development of metastatic potential in these patients. It is likely that metastatic trigger events are rare and arise stochastically, which means it will be difficult to predict the lineage that will eventually metastasize.

Metastatic potential appeared to develop twice in two patients. In A34, the lineage that metastasized to the liver diverged from the intra-prostatic lineage at a higher position (nearer the root node) than the spinal cord metastases that were removed 11 years prior to death. In A24, the cancer in the sigmoid colon serosal sample was not from the same lineage that led to the systemic metastases. As A24’s pelvis was treated with radiotherapy, it appears that this treatment eliminated the metastatic potential of this lineage. The substantial amount of time to death after these lineages branched in the prostates of these patients (at least 11 and 10 years respectively) provides further support that evolution of metastatic potential is a rare event.

Both tissue and liquid biopsy may be of limited utility in assessing overall metastatic status. Information available from tissue biopsies will be incomplete, due to the presence of multiple intra-prostatic lineages, and there is currently no means to identify which lineage is most likely to metastasize. However, the fact that evolutionary branching usually precedes metastatic seeding means that heterogeneity could be used as a marker for advanced disease. This may explain the findings of a previous study, which showed that subclonality in the prostate is indicative of poor prognosis^42^. The ctDNA detected in the blood did not give a full representation of extant metastatic lineages, but it did display subclones corresponding to the dominant lineage in all cases. It may have been possible to identify cells from more metastatic sites using more sensitive analysis of individual circulating tumor cells^43^. Nonetheless, the variable representation of metastatic subclones does indicate that the circulating systemic tumor burden only originates from a subset of metastatic sites. In two cases (A24 and A34) the disappearance of a lineage found earlier in the blood appears to be a true indicator of therapeutic response, which could be useful in designing clinical trials aimed at evolutionary steering of tumors to beneficial outcomes. The discovery of metastatic lineages in CSF not found in ctDNA indicates that a combination of CSF and blood liquid biopsies could provide a more accurate assessment of metastatic status in prostate cancer patients. Our study strongly indicates that analysis of autopsy and/or multiple metastases together with liquid biopsy results will be critical to determining when and how liquid biopsy should be utilized in general clinical practice.

The 10 men who are the subjects of the study received treatment for prostate cancer between 1987 and 2005 (Supplementary Figs. 1–10). These ranged from drug-free (radiotherapy and orchidectomy in A32) to multiple androgen-manipulating and chemotherapeutic agents varying both in type and intensity. The standard of care for prostate cancer has changed in the intervening years. However, many of the drugs they received, such as leuprolide and flutamide, are in the same class as currently used drugs. It is remarkable that despite differences in treatment received, they all showed similar evolutionary patterns in the prostate and metastatic periphery as reported above. Therefore we believe the results will be relevant to men treated with modern therapies, and this cohort of deeply studied men can serve as a benchmark for comparison of tumor evolution under selective pressures applied by newer pharmaceutical agents in future studies.

A detailed picture of the evolution from primary to metastatic prostate cancer is now emerging. Considerable intra-tumoral heterogeneity is commonly acquired from several years of growth within the prostate, extending through local invasion into the seminal vesicles and bladder. Selection, frequently acting at the genetic level, enables cells from a single lineage to escape the prostate and colonize distant sites. This seeding event is followed by the re-emergence of heterogeneity, albeit just within the metastatic lineage, which is rapidly distributed across multiple sites through polyclonal migration.

## Methods

### Study materials

Tissue samples were collected as part of the PELICAN integrated clinical-molecular autopsy study of lethal prostate cancer. The 10 patients included in this study consented to participate in the John Hopkins Medicine IRB-approved study between 1995 and 2005. The mean age of the study subjects at the time of diagnosis of prostate cancer was 61 years. Samples studied were from surgical samples, body fluid, and autopsy samples collected at multiple time points between 1987 and 2005. A summary of patients, samples, and targeted regions included in the study is shown in Table 1. Additional characteristics of the study patients, including clinical timelines and samples are contained in Supplementary Figs. 1–10, Supplementary Data 2 and 3, and Gundem et al.^11^ supplementary information.

**Table 1:** Summary of patients and samples included in the study.

Study patient	A10	A12	A17	A21	A22	A24	A29	A31	A32	A34
Years between diagnosis and death from metastatic prostate cancer	4	4	4	5	2	11	15	8	9	12
No. target subs	675	266	958	959	2258	703	362	714	1733	1094
No. targeted indels	147	72	163	124	233	209	59	115	263	234
Total no. of mut. analyzed	728	309	870	850	2032	509	466	292	683	1133
No. samples in Gundem et al.^11^	4	3	5	8	10	4	2	5	5	4
No. samples in current study	10	19	14	19	15	22	6	18	16	11
No. of sample time points	2	3	3	2	3	3	3	3	4	3

Additional details in Supplementary Data 2 and 3.

A total of 163 samples, including 37 body fluid samples (including whole blood, plasma, serum, CSF, pleural fluid, pericardial fluid, and urine), 53 high molecular weight (HMW) samples from microdissected frozen tissue (new aliquots of same DNA used in Gundem et al.^11^), and 73 laser-microdissected paraffin-embedded (PE) tissue samples were included in the study.

Body fluid samples obtained from the 10 men during life and at autopsy are named according to source (serum, plasma, whole blood, CSF, pleural fluid, pericardial fluid, and urine), and sampling days prior to death (dptd) from prostate cancer.

PE tissue samples were from prostate biopsy, radical prostatectomy, transurethral resection of the prostate (TURP), from prostate removed at autopsy, or from other surgery as indicated in Supplementary Data 2. PE tissue samples were either formalin-fixed paraffin-embedded (FFPE) or alcohol-fixed paraffin-embedded (AFPE) with ethanol/methanol fixative (3:1 EM)^44^ as indicated (Supplementary Data 2). One laser-microdissected FFPE autopsy sample (A10-20322-BladderInfiltMET) is also included in the current study. PE regions of interest (ROI) in available tissue sections were chosen based on anatomic location and morphology, including tumor areas of variable morphology, noncancerous areas as technical controls to test for DNA cross contamination in paraffin tissue, benign prostatic hypertrophy (BPH), and some tiny prostate intraepithelial neoplasia (PIN) regions. Multiple regions of a primary prostate cancer were laser-dissected from patients A24, A31, A32, and A34 samples. In total, prostate samples from at least one-time point (biopsy, radical prostatectomy, transurethral resection, or autopsy) were analyzed for all 10 patients. Images of laser-dissected regions in PE tissue are contained in Supplementary Data 3.

Autopsy body fluid samples were collected using needle aspirations of blood from the right ventricle and CSF from the 4th cerebral ventricle using sterile methods. Because postmortem clotting separates clotting components and many nucleated cells from the remainder of the blood, liquid blood removed from the right ventricle at autopsy is referred to as “whole blood” but is typically lower in cell content than whole blood removed during life. Urine collected during life was collected by clean-catch midstream sampling, while urine collected at autopsy was collected by sterile suprapubic bladder aspiration. PE tissue blocks were stored at room temperature and body fluid samples were frozen at −80 °C prior to DNA isolation.

Patients’ detailed clinical histories were obtained from medical records curated from all available hospital and clinic records and in some cases the patient’s own diaries provided directly to the study team. Time from prostate cancer diagnosis to death ranged between 2 and 15 years. In six men, the microdissected prostate material studied (frozen, FFPE and 3:1 E:M fixed) was removed at autopsy, and the other four patients (A12, A17, A21, and A24) underwent radical prostatectomy soon after the initial diagnosis of prostate cancer and FFPE material from these men was laser-microdissected and studied. In addition, prostate biopsy samples from patients A17, A22, A24, and A29 were laser microdissected and included in the study.

We also analyzed a frozen spinal cord root-compressing sacral bone metastasis sample surgically removed 11 years prior to death from patient A34 (A34-21408), 224 days (7.5 months) after biopsy diagnosis, and laser-dissected FFPE TURP samples removed due to bladder outlet obstruction from patients A10 and A34. Blood samples taken prior to death were collected from A12 (46 dptd), A22 (448 dptd), A31 (1186 dptd), A32 (1040 dptd and 20 dptd) and A34 (4040 dptd). Blood samples taken during autopsy are available for A10, A12, A17, A22, A24, A31, A32, and A34. Other liquid samples, e.g. urine and CSF, were analyzed for A17 (CSF from autopsy), A29 (urine from 409dptd and autopsy and CSF from autopsy), A31 (urine from 675 dptd and autopsy) and A32 (urine from 655 dptd and CSF from autopsy).

### Sample tracking

A laboratory database system was used to maintain traceability of each DNA sample from the time of autopsy, through cryostat or laser microdissection (LM), analyte isolation, storage, shipping, assays, analysis, and EGA data repository submission as described below.

### Cryostat microdissection and DNA isolation (frozen tissue samples)

Tissue blocks embedded in OCT (Optimal Cutting Temperature compound, Tissue-Tek) were serial −20 °C cryostat microdissected for histological tumor purity >75%. During serial dissection, prior to isolation of sections for DNA extraction, OCT compound was trimmed away completely. HMW DNA was isolated from 50 to 500 six micron sections per dissected tissue block using proteinase K digestion and phenol/chloroform extraction.

### LM and lysate preparation (PE tissue samples)

For each tissue block studied, 4 µm face sections were stained with hematoxylin and eosin (H&E), scanned with Aperio whole slide scanner, and ROI in each digital image were annotated for dissection (Supplementary Data 3). For cases A10, A12, A17, A21, and A22, 5 µm sections on glass slides were microdissected. For cases A24, A29, A31, A32, A34, 7 µm thick serial sections mounted on framed PEN membrane slides (Molecular Machines and Industries) were microdissected. Just prior to microdissection, slides were incubated in xylene (5 min.x2), dehydrated in 100% ethanol (1 min.x2), stained with cresyl violet acetate and eosin mix^45^ for 30 s, dehydrated in 100% ethanol (30 s.x2), cleared in xylene (3 min.x2), dried in fume hood for 5 min and kept in a desiccator for 1 h prior to LM. ROI were dissected from membrane slides by laser cutting on MMICellCutPlus (Molecular Machines & Industries) instrument, and from archival glass slides by manual microdissection of laser annotated ROI using a Leica MZ8 dissecting microscope^46^.

### Lysate preparation (PE samples)

Lysates containing DNA were generated from microdissected tissues using PicoPure DNA Extraction Kit digestion buffer (Thermo Fisher) by incubating samples at 56 °C for 68 hours with the addition of 10 µl of 20 mg/ml Proteinase K (Thermo Fisher) every 24 h. Samples were then heated to 95 °C for 10 min to inactivate Proteinase K.

### Isolation of DNA from body fluids

QIAamp circulating nucleic acid kit 55114 (Qiagen) was used to isolate DNA from serum, plasma, CSF, and urine. QIAamp DNA blood midi/maxi kit 51185 (Qiagen) was used to isolate DNA from whole blood, and pleural fluid. Both kits were used following manufacturer’s protocols.

### Double strand library preparation (frozen and body fluid samples)

12.3 to 96.1 ng of gDNA, quantified with Qubit dsDNA BR Assay kit (Thermo Fisher Scientific), was fragmented with Episonic Multi-Functional Bioprocessor 1100 (Epigentek Group Inc., Farmingdale, NY, USA). The libraries were created with Thruplex DNA-seq 96D kit (Rubicon Genomics) according to manufacturer’s protocol. 10–50 ng of DNA was used for library preparation. Five amplification cycles were used. The amplified libraries were purified with 50 µl of NucleoMag NDS Clean-up and Size Select beads and eluted to 30 µl of PCR grade water. The libraries were quantified with LabChip GX HT DNA HiSens chip (PerkinElmer, Waltham, MA, USA) (Supplementary Data 2).

### Single strand library preparation (PE samples)

Five to 150 ng of lysate gDNA was quantified with Qubit dsDNA BR Assay kit (ThermoFisher, Waltham, MA, USA) and fragmented with Episonic Multi-Functional Bioprocessor 1100 (Epigentek Group Inc., Farmingdale, NY, USA). The libraries were created with Accel-NGS 1S Plus DNA Library Kit (Swift Biosciences, Ann Arbor, MI, USA) according to manufacturer’s protocol. Four to 40 ng of DNA were used for library preparation. Seven amplification cycles in PCR were used for all the samples. Agencourt AMPure XP beads (Beckman Coulter, Brea, CA, USA) in 1.8 times the reaction volume were used for purifications. 1S Plus Dual Indexing Kit was used for all the samples. The libraries were quantified with LabChip GX HT DNA HiSens chip.

### Custom capture target selection

From the whole-genome sequencing (WGS) data (based on 51 fresh frozen samples) produced in Gundem et al.^11^, we selected a subset of 12257 variants (substitutions and indels) representative of the phylogenetic evolution in 10 metastatic prostate cancer patients. In the selection process, first, the mutations located in UCSC repetitive regions were excluded. Then, a random subset of the variants was sampled from each mutation clone (a cluster identified by the Dirichlet Process clustering algorithm, i.e., a branch on a phylogenetic tree) representing each clone. The resulting random subset contained 11,135 variants. The final set of targeted mutations additionally included all 1122 protein-altering variants producing the targeted set of 12,257 variants. Targeted regions for sequencing were produced by adding 110 nucleotide flanks to each indel target and flanks of 60 nucleotides were added to the substitution target positions. A greater targeted area for indels was chosen to avoid false-positive indel calls due to misalignment. To prepare the final set of targeted regions, all overlapping regions (*n* = 370) were merged, resulting in 11,886 regions that covered 1.58 Mb. This set of targeted regions was used in the Nimblegen probe design, which produced 12,445 baits (580 targeted regions were covered by 2 baits, with 13 baits covering 2 target regions). Overall targeted genomic regions and targeted coding indels and coding substitutions are listed in Supplementary Data 4–6.

### NimbleGen SeqCap EZ custom capture

Four to 15 samples were pooled to each capture reaction. Equal nanograms of each library were in each pool (13–40 ng per sample). The capture was performed according to SeqCap EZ Library SR User’s Guide v5.0 protocol with the following exceptions using SeqCap EZ Choice Library (Roche Sequencing, Pleasanton, CA, USA). For the test samples HE oligos were used and for the dual index libraries 1 µl per xGen Universal Blocking oligo Truseq HT-i7 and i5 (Integrated Device Technology, San Jose, CA, USA) were used instead of the HE oligos. Ten cycles were used for post-capture amplification instead of 14. The captured and amplified pools were quantified with 2100 Bioanalyzer High Sensitivity DNA Analysis Kit (Agilent Technologies, Santa Clara, CA, USA).

### Sequencing

The libraries were sequenced in HiSeq1500 and HiSeq2500 instruments using paired-end 101 cycle V4 high output chemistry and paired-end 101 cycle V2 Rapid run chemistry (Illumina, San Diego, CA, USA). Targeted sequencing data was obtained from 150 of 163 total samples collected from the 10 patients with an average coverage of 785.7× (range 64.9–2253.2), as described in the Sample Qualification section below and in Supplementary Data 2.

### Preprocessing and alignment of sequencing data

Bioinformatic processing was performed following an established protocol^47^. Specifically, raw reads were corrected for low base-calling quality, Illumina adapters, and short length using Trimmomatic^48^ (with parameters: leading = 3, trailing = 3, sliding window = 4:15, illumineclip = 2:30:10, and minlen = 36). Quality trimmed paired-end reads were aligned to the human reference genome build GRCh38 with BWA-MEM^49^ and alignment files sorted by coordinate and marked for duplicates with Picard tools^50^. The Genome Analysis ToolKit (GATK)^51^ tools were then run to perform base quality recalibration and realignment of indels. Finally, alignment files from the same biological sample were merged and duplicate reads were remarked using Picard tools^50^. Quality control analysis was performed using FastQC^52^ with default settings. The GATK resource bundle files used in the process were converted from GRCh37 to GRCh38 using CrossMap^53^ and chain files downloaded from EnsEMBL. Default parameters were used unless specified otherwise.

### Variant calling

For variant calling, the last two bases from each alignment were removed using the trimBam function of the bamUtil^50^ tool. Each tumor sample was then paired with its patient-matched control sample that had been WGS earlier in Gundem et al.^11^ and variants were called using GATK MuTect2^51^. Default parameters were used, except that down sampling and filtering of duplicate reads were disabled and clustered_events flags were cleared to utilize all deep-sequenced coverage data. Finally, variants were filtered against a panel of normals generated from samples from healthy patients, and by discarding those that were not within panel’s target regions +/− 150 bp. De novo mutation calling was performed, and SNVs were used in the analysis if they were called separately in two or more samples.

### Sample qualification

Summary qualification results for 163 samples entering the sequencing process are contained in Table 2, and are detailed in Supplementary Data 2, 3, and 7. Seven body fluid and six microdissected PE samples failed library preparation, most likely due to DNA fragmentation and low DNA concentration. For the remaining 150 samples from which data were analysed, samples are divided into EGA datasets A (118 samples) and B (32 samples). Samples in EGA dataset A are illustrated in the main figures and are grouped together to illustrate the main findings. Some of the body fluid samples in EGA dataset A contain low cancer variant signal and are included as comparison to later samples from the same patient with strong signal in body fluids. A29-21276-AutCSF data displayed evidence of possible contamination by extraneous human DNA and was excluded from the anaysis, but is included in EGA dataset A as there are still observable tumor variants irrespective of contamination. The 32 samples in EGA dataset B containing low signal or ancillary findings and are accessible on EGA for researchers who may wish to further examine them.

**Table 2:** Sample summary.

Samples	Entering study	Failed library prep	EGA dataset A	EGA dataset B
Body fluid	37	7	25	5
	(Serum 12, plasma 7, whole blood 5, CSF 3, urine 6, pericardial fluid 2, pleural fluid 2)	(Plasma 1, urine 2, pericardial fluid 2, pleural fluid 2)	(Serum 12, plasma 6, whole blood 4, CSF 2)	(Whole blood 1, urine 4)
Frozen tissue	53	0	52	1
	(Metastatic PCa 47, primary PCa 5, kidney NL 1)		(Metastatic PCa 47, primary PCa 5)	(Kidney NL 1)
Paraffin embedded (PE) tissue	73	6	41	26
	(Metastatic PCa 9, primary PCa 42, normal prostate 18, normal lymph node 1, BPH 2, PIN 1)	(Metastatic PCa 1, primary PCa 4, normal prostate 1)	Metastatic PCa 8, primary PCa 33)	(Primary PCa 5, noncancerous prostate and lymph node 21)
Total	163	13	118	32

Sequence data from 150 samples where library creation succeeded were analysed and are submitted to EGA. 118 samples central to findings presented in the main text are contained in EGA dataset A. Data from 32 samples with important background control and other findings are included in EGA dataset B and are discussed in the Sample Qualification section and detailed in Supplementary Data 2, 3 and 7.

The results from samples in EGA dataset B are illustrated in Supplementary Data 7 and include 5 body fluid samples (A22-21268-AutClottedBlood, A29-21274-409dptdUrine, A29-21275-AutUrine, A31-21279-675dptdUrine, and A31-21283-AutUrine). A22-21268-AutClottedBlood exhibited a background noise pattern. The four other body fluid samples in EGA dataset B are urine samples, one of which contains cancer variant signals similar to portions of the primary tumor (A31-21283-AutUrine). The other three urine samples (A29-21274-409dptdUrine, A29-21275-AutUrine, A31-21279-675dptdUrine) show mainly a noise pattern. These data are interesting and indicate that DNA from primary prostate cancer can be detected in urine at times, but the data are limited to only one positive and three samples that show mostly noise and are left out of the main findings for brevity.

The one frozen tissue noncancerous sample in EGA dataset B (A21-21091-LKidneyNL-PD12337b) is a technical control.

For the 67 microdissected PE samples included in the study, 21 noncancerous controls were included. PE samples are infused with several liquids during collection and processing, making them potentially more susceptible to pre-sectioning DNA cross-contamination than frozen tissue samples. Seventeen of 21 PE samples considered to be noncancerous prostate control samples prior to laser dissection showed no evidence of cancer DNA variant signal. Results from these samples show what we interpret as noise (<50% SNVs with CCF > 0 in a cluster) in terms of detected cancer-specific variants, as illustrated in clustering figures shown in Supplementary Data 7. These 17 samples are A12-20325-LJPNLGLANDS, A12-20326-LJPSTROMANL, A12-20329-LNCNLgerminal+mantle, A12-20330-RGP-1PIN, A12-20331-RGP-1BPHglandsNL, A12-20332-RGP-1StromalSpindleNodule, A12-20333-RGP-1PeriphZoneNL, A17-20340-1F1LN2NL, A21-20347-3RHPPIN, A21-20348-3RHPBPH, A24-20443-PINAreaA, A24-20447-PINAreaB, A24-20449-BiopsySite, A24-20450-ProstateInkedMarginA, A24-20451-NontumorAreaA, A24-20452-NontumorAreaB, and A24-20458-ProstateInkedMarginB.

Four PE samples (A10-20321-TURPNL, A29-20462-BxNoncancerousEpithelium, A31-20470-NontumorMuscle, A34-20479-NontumorStroma) were defined as noncancerous prior to laser dissection and show cancer variant signals in cluster diagrams shown in Supplementary Data 7. Pathology review of the whole slide image ROIs for these samples shows that these samples are likely to contain small numbers of cancer cells detected by the deep targeted sequencing performed in this study.

In A10-20321-TURPNL noncancerous ROI dissected from 8 TURP chips are in close proximity in many areas to high-grade infiltrative cancer. The presence of subclones 12–16, also present in the A10-20320-TURPCAGL5 from this patient, is not surprising. The portions of 8 TURP tissue chips dissected each has close proximity to high-grade prostate cancer regions in the same TURP chip, and it is likely that when these regions were dissected from adjacent glass slides, some cancer cells were included. Cross-contamination of the sample after extraction from A10-20320-TURPCAGL5 to A10-20321-TURPNL is ruled out because of the presence of subclone cluster 9 in 20320 that is absent from 20321.

In A29-20462-BxNoncancerousEpithelium, the cancer variant cluster data from this sample shows a low-level cancer variant signal. Review of the ROI selected for laser dissection shows four foci of suspicious cells likely to be prostate cancer that was not appreciated prior to the dissection. Our interpretation is that this faint signal comes from a small fraction of tumor cells sequenced at high depth. Note that the tissue block dissected in this case had regions of cancer in it that had disappeared when deeper sections were cut, so it is not unexpected that some cancer cells are in the ROI and likely in the adjacent dissected slides.

In A31-20470-NontumorMuscle, the right side of the defined ROI selected for laser dissection comes within 100 microns of visible cancer cells. Since the cancer variant cluster figure shows a faint primary cancer signal in this sample, it seems most likely that the regions dissected from adjacent slides contained a small amount of cancer.

A34-20479-NontumorStroma is similar to A10-20321-TURPNL in that it also is from a TURP sample with a large amount of surrounding high-grade cancer. In addition, the right side of the single TURP chip dissected for this sample has two tiny foci of suspicious cells that likely account for the faint cancer signal in the cluster figure for this sample.

Of 42 primary prostate cancer regions dissected from PE tissue, 5 gave scant signal consistent with low quality or quantity of DNA and these are included in EGA dataset B. All five are from tissues that were formalin-fixed. These are A21-20346-3RHPCAGL3, A21-20349-3RECAGL3, A21-20350-3RECAGL4fusedpapillary, A21-20356-3LCCAGL3, and A24-20461-BxNontumorandtinyquestionableareas. The latter sample (20461) is from a tiny partially crushed prostate biopsy region and does show signal above background consistent with primary tumor, but we judge the signal too limited to make firm conclusions about evolution.

### Tumor cellularity estimation

For the 51 samples that were previously sequenced with WGS, we used the WGS-based purity estimates generated with the Battenberg algorithm (https://github.com/cancerit/cgpBattenberg). For the 97 new samples sequenced with targeted sequencing, tumor cellularities were estimated using a combination of two methods: based on the variant allele frequency information and the Dirichlet Process (DP) clustering algorithm. The initial estimates were calculated as the mode of the Gaussian kernel density function fitting the distribution of VAFs of truncal mutations for each sample. VAFs lower than 1% were excluded from this estimation, as they likely correspond to false-positive variant calls. All samples were considered to be diploid, except for the samples from A31 and A32, which were treated as tetraploid based on the average sample ploidy estimated by the Battenberg algorithm in Gundem et al.^11^. A subsequent fine-tuning of the estimated sample tumor cellularities was done based on the data from the Dirichlet Process clustering algorithm as in Gundem et al.^11^. The cellularity values were modified so to achieve values of CCFs approximately equal to 1 (between 0.75 and 1.25) in the truncal cluster produced by the DP clustering algorithm.

### Copy number estimation

CNA in the studied samples were estimated using CNVkit^24^ to estimate copy number profiles using the off-target reads in our targeted sequencing data. To construct pooled copy number references required by CNVkit in the read count normalization, we used samples with the estimated CCF lower than 1%, i.e., non-tumor samples. Body fluid samples not containing traceable amounts of cancer cells were used in the reference for both tumor containing body fluid and HMW fresh-frozen tissue samples. For the PE tumor samples, non-tumor FFPE samples were used in the reference. To produce final copy number profiles, log-ratios provided by the CNVkit were converted into clonal copy number estimates with correction for sample’s CCF and ploidy using a formula as in Adalsteinsson et al.^54^. For the FFPE and BF samples the ploidy values to be used in the copy number estimation were obtained by comparing the CNVkit copy number profiles to the copy number profiles of the HMW samples obtained with the Battenberg algorithm. Only clonal copy numbers from the HMW copy number profiles were used in the comparison. To estimate the correct ploidy value for each sample we explored a range of possible ploidy values, from 2 to 5 with step of 0.1. The ploidy value producing the highest match between the estimated copy number profile with one of the copy number profiles of the HMW samples of the same patient was selected. As the majority of the analyzed samples were obtained at the earlier time points compared to the WGS analyzed autopsy samples, the mismatching copy numbers were considered to be correctly estimated if they were equal to 2, i.e., corresponding to the copy neutral regions.

As a quality control we compared copy number profiles of the HMW samples estimated with CNVkit from the targeted sequencing data and Battenberg from the WGS data. In the comparison there were three outliers due to a big difference between ploidy values used in the copy number estimation, i.e. sample-specific in the Battenberg and patient-specific in the CNVkit estimations. The patient-specific ploidy values used by CNVkit were based on the majority of the rounded ploidy values from the WGS HMW samples analyzed in Gundem et al.^11^ with Battenberg. The three outliers are A12-c, A21-f, and A29-a with their Battenberg and CNVkit specific ploidies correspondingly: 4.47 vs 2, 3.76 vs 2, and 4.92 vs 2.

Excluding the three outliers mentioned above, the percentage of the bp in the autosomes with the same copy number estimated by both methods ranged from 46.24 to 93.11% with the mean at 78.13% and median at 83.13%. The comparison was performed only in the autosomes as both methods produce less reliable estimations in the sex chromosomes.

### CCF estimation

The CCF was calculated by adjusting the variant allele frequencies with copy number and tumor purity following the method in Bolli et al.^21^. In brief, the mutation copy number, *n*_mut_, equivalent to the fraction of tumor cells carrying a given substitution multiplied by the number of chromosomal copies of the mutation, *n*_chr_, is given by

$$n_{{\mathrm{mut}}} = f_s\frac{1}{\rho }\left\{ {\rho n_{\mathrm{locus}}^t + n_{{\mathrm{locus}}}^n\left( {1 - \rho } \right)} \right\},$$

where *f*_*s*_ is the fraction of mutated reads observed in the sequencing data, and *ρ*, *n*^*t*^_locus_ and *n*^*n*^_locus_ are respectively the tumor purity, the locus-specific copy number in the tumor cells and the locus-specific copy number in the normal cells. For all mutations in amplified regions with major copy number *C*, the observed number of mutant and wild-type reads was compared against the expected *f*_*s*_ values resulting from the mutation being present on *1, 2, …, C* chromosome copies, assuming a binomial distribution, and *n*_chr_ was assigned the value of *C* with the maximum likelihood. CCF was then calculated as *n*_mut_*/n*_chr_.

SNVs in fixed samples were only retained if found in CNV regions called with CNVkit that agreed with those called by the Battenberg algorithm in at least one HMW sample.

### Mutation clustering

The Dirichlet Process clustering (DPClust) method^21^ was used to group mutations present at similar CCF in each tumor sample. DPClust was performed for each patient on the samples with estimated tumor cellularity above 15%. The DP clustering corresponded to a model defining mutations as originating from an unknown number of subclones contributing to a tumor in unknown proportions and represented by an unknown fraction of tumor cells. To perform the analysis, we used the DPClust package available from Github (https://github.com/Wedge-Oxford/dpclust) version 2.2.5, run for 10,000 MCMC iterations, of which 9000 were burn in. Other parameters were conc_param = 1, cluster_conc = 5, density.smooth = 0.001, max.burden = 1.5. The output of DPClust is a set of subclones, each of which has an associated CCF within each sample analyzed and the number of mutations associated with that subclone (Supplementary Data 1).

### Phylogenetic tree construction

Patient-specific phylogenetic trees incorporating mutation clusters identified by DPClust were manually constructed using the Sum and Crossing Rules^22^. In brief, the sum rule operates upon the premise that if the CCFs of two mutation clusters in any sample add up to more than the CCF of their shared ancestral cluster, they must be collinear. The crossing rule states that if two mutation clusters B and C are descendants of mutation cluster A, and if cluster B has higher CCF than cluster C in one sample and cluster C has higher CCF than cluster B in another sample, clusters B and C must be branching. Underlying the Sum and Crossing rules is the Infinite Sites Assumption, which states that each mutation has occurred only once. In practice, a very small number of positions in the genome may be subject to mutation more than once, but this occurrence is sufficiently rare that it does not impair our ability to define mutation clusters. In general, the sum and crossing rules do not restrict the space of possible trees to a single candidate. However, when applied simultaneously to the CCFs of mutation clusters across multiple samples they severely constrain the space of possible trees. In cases where branched or sequential assignments could not be definitively distinguished, which only occurred in the leaves of the trees, we followed the corresponding tree obtained from WGS in Gundem et al.^11^. Clusters with a mean CCF < 0.02 or containing over 50% indels were not included in tree construction.

### Putative drivers on chromosome 8p

We report gene names of known drivers on 8p in Fig. 4. These were defined as those genes listed on Cancer Genetics Web chromosome 8 (http://www.cancer-genetics.org/clinkc08.htm, accessed 25 March 2020), for which the *Implicated In* field included the term *Prostate Cancer*, and were found within region 19,962,013–27,489,421 on 8p according to UCSC Genome Browser^55^ (http://genome.ucsc.edu/, accessed 25 March 2020), with GRCh38 assembly.

### Chronological timing

Mutational signature 1^25^ is known to represent a clock-like process as it is generated at relatively constant rate^56^. We used WGS SNV calls derived from fresh frozen samples from Gundem et al.^11^ to identify sets of point mutations that corresponded to the trunk until the first intra-prostatic branch, between the first branch and the first metastatic seeding event, and then from seeding until sampling occurred. The non-negative matrix factorization method used to identify mutational signatures is susceptible to errors when mutational counts are low, as may be the case in some of our tree segments. As signature 1 consists of C > T mutations in a CpG context, we circumvented this issue by using raw counts of these mutational types as a proxy for signature 1. These were generated based on the DPClust output where each single nucleotide variant from a patient was assigned to a subclone. For each variant in each subclone, the Biostrings (version 2.48.0) and BSgenome (1.48.0) Bioconductor packages in R (version 3.5.1) were used to retrieve the 5’ and 3’ adjacent bases of the mutation based on the mutation coordinate. A count was kept of C > T mutations in a CpG context on either the forward or reverse strand.

### Reporting summary

Further information on research design is available in the Nature Research Reporting Summary linked to this article.

## Supplementary information

Supplementary Information

Description of Additional Supplementary Files

Supplementary Data 1

Supplementary Data 2

Supplementary Data 3

Supplementary Datat 4

Supplementary Data 5

Supplementary Data 6

Supplementary Datat 7

Reporting Summary

## Data Availability

The raw targeted sequencing data have been deposited in the European Genome Archive repository under the accession codes EGAD00001005381 and EGAD00001005382 (TenMenDeep EGA Datasets A and B respectively). The whole genome sequence data referenced in this study are available from the European Genome Archive repository under accession code EGAD00001000891 and is available on request. The gene name data referenced during the study are available in a public repository from the UCSC Genome Browser website (chromosome 8, GRCh38) [https://genome.ucsc.edu]. All the other data supporting the findings of this study are available within the article and its supplementary information files and from the corresponding author upon reasonable request. A reporting summary for this article is available as a Supplementary Information file.

## Code availability

Battenberg algorithm is available at https://github.com/cancerit/cgpBattenbergDPClust is available at https://github.com/Wedge-Oxford/dpclust
