# Supplementary material for: Prostate cancer evolution from multilineage primary to single lineage metastases with implications for liquid biopsy
Source: Nat Commun. 2020 Oct 8;11:5070. doi: 10.1038/s41467-020-18843-5 (PMC7545111; doi:10.1038/s41467-020-18843-5)
Supplement: Supplementary file 1 — Supplementary Information [file 41467_2020_18843_MOESM1_ESM.pdf]

SUPPLEMENTARY INFORMATION FOR

# Prostate cancer evolution from multilineage primary to single lineage metastases with implications for liquid biopsy

Woodcock et al.

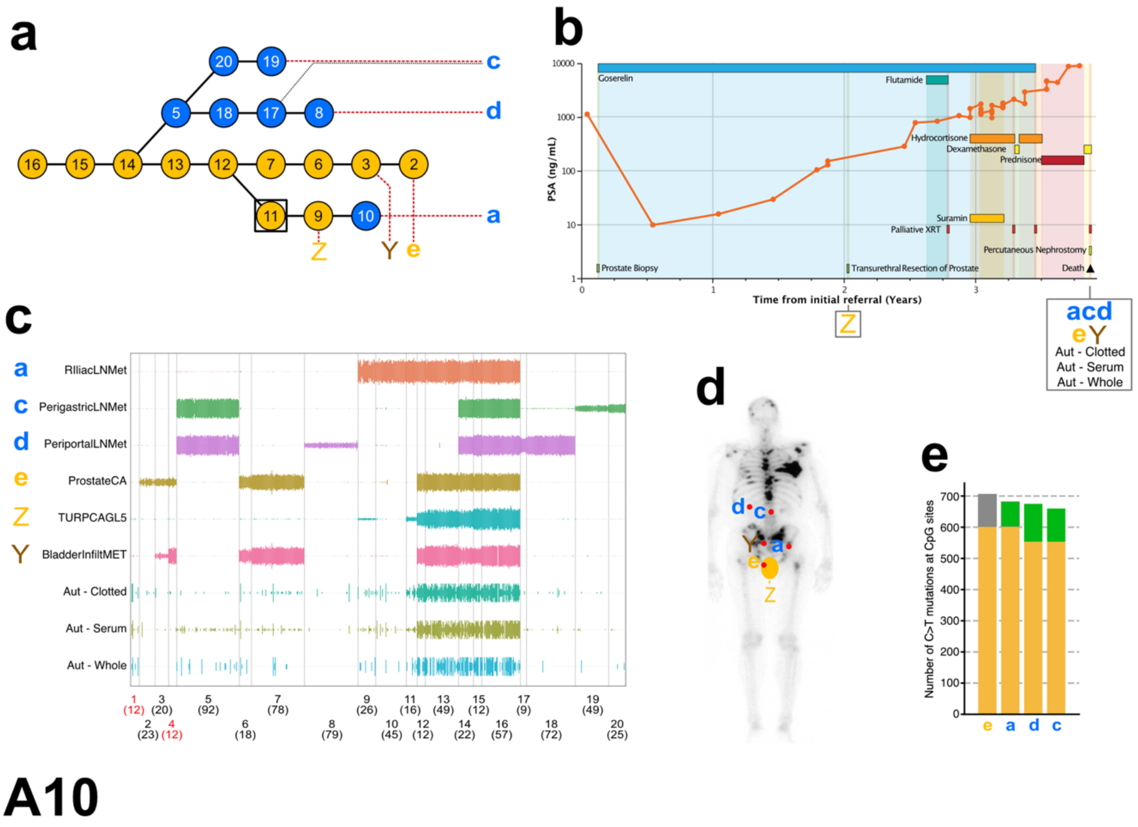

Supplementary Figure 1 | Ancillary information for patient A10.

**Supplementary Figure 1 | Ancillary information for patient A10. a)** The phylogenetic tree (as in MP Fig. 1.) showing the subclonal cluster numbers identified with DPClust, displayed in subfigure **c**. Subclonal clusters are coloured as in MP Fig. 1. **b)** The clinical timeline from diagnosis to death showing PSA levels (red line, scale on y-axis) and treatments as annotated. The time of collection of samples is shown by the corresponding sample letter/name underneath the x-axis. **c)** The subclonal clusters identified by DPClust (x-axis), and the estimated cancer cell fraction (CCF) of each SNV (each individual bar) for the tumour in each of the samples (y-axis). Samples taken from body fluid were scaled so the CCF of the largest subclonal component was equal to 1, and the subfigure displays clipped values (at CCF=1) as SNV abundance displayed high variance. The numbers under the x-axis show the cluster number, and the number of SNV/indels assigned to the cluster in brackets. Numbers in red were not used to construct the phylogenetic tree as they contained >50% indels (see Methods). Original sample names are shown on the y-axis, along with sample letters used throughout this study. Body fluid samples denoted by time of sample - Aut: Autopsy; and body fluid type - Whole: Whole Blood. See Supplementary Data 2 and Methods for more details on samples. **d)** Bone scan showing the approximate anatomical locations from which samples were taken, as indicated by their corresponding sample letter. **e)** Total number of C>T mutations in a CpG context observed in fresh frozen samples for patient A10, split into mutations accumulated from birth to first intra-prostatic branch or metastatic seeding event (yellow bar), and mutations accumulated from first branching event (grey bar) or metastatic seeding (green bar) until death. See Methods for more details. In this patient, the intra-prostatic branch that leads to the lineage ending at subclone 2 occurred at subclone 12, as did the metastatic lineage ending at subclone 10. Metastatic lineages ending at subclones 19 and 8 diverged from the intra-prostatic lineage at subclone 14.

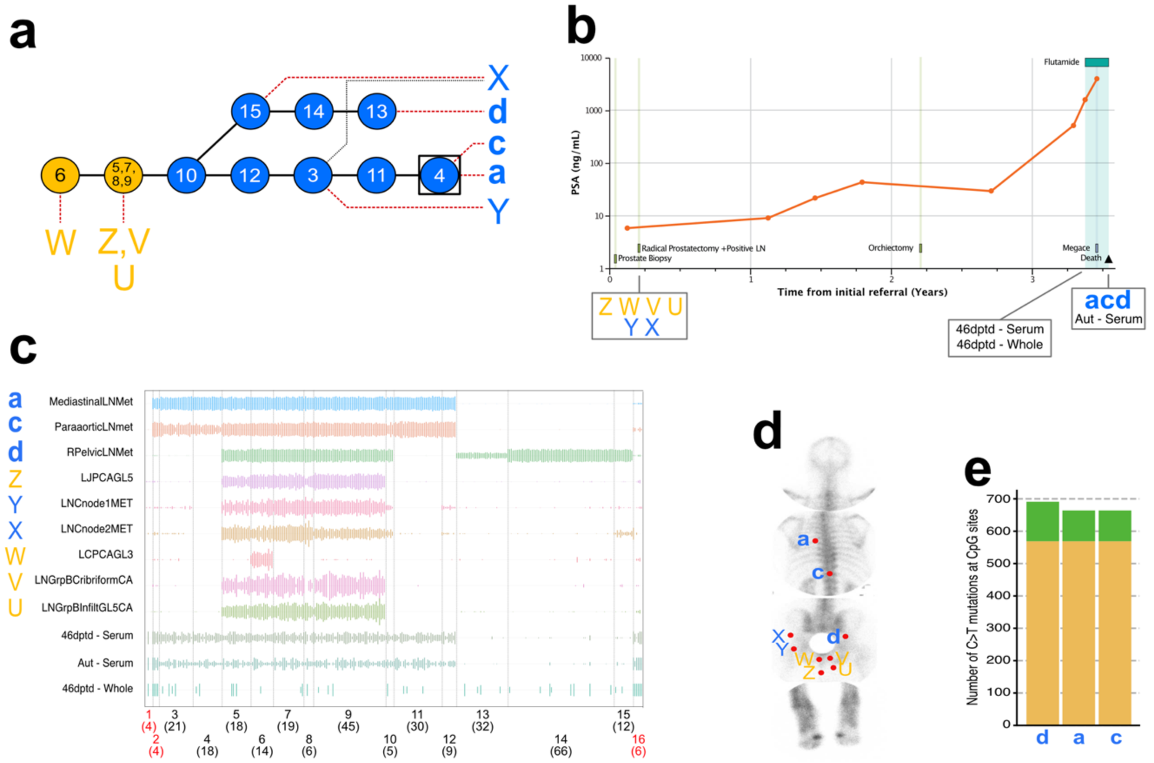


**Supplementary Figure 2 | Ancillary information for patient A12. a)** The phylogenetic tree (as in MP Fig. 1.) showing the subclonal cluster numbers identified with DPClust, displayed in subfigure **c**. Subclonal clusters are coloured as in MP Fig. 1. **b)** The clinical timeline from diagnosis to death showing PSA levels (red line, scale on y-axis) and treatments as annotated. The time of collection of samples is shown by the corresponding sample letter/name underneath the x-axis. **c)** The subclonal clusters identified by DPClust (x-axis), and the estimated cancer cell fraction (CCF) of each SNV (each individual bar) for the tumour in each of the samples (y-axis). Samples taken from body fluid were scaled so the CCF of the largest subclonal component was equal to 1, and the subfigure displays clipped values (at CCF=1) as SNV abundance displayed high variance. The numbers under the x-axis show the cluster number, and the number of SNV/indels assigned to the cluster in brackets. Numbers in red were not used to construct the phylogenetic tree as they contained >50% indels (see Methods). Original sample names are shown on the y-axis, along with sample letters used throughout this study. Body fluid samples denoted by time of sample - Aut: Autopsy, dptd: days prior to death; and body fluid type - Whole: Whole Blood. See Supplementary Data 2 and Methods for more details on samples. **d)** Bone scan showing the approximate anatomical locations from which samples were taken, as indicated by their corresponding sample letter. **e)** Total number of C>T mutations in a CpG context observed in fresh frozen samples for patient A12, split into mutations accumulated from birth to the first metastatic seeding event (yellow bar), and mutations accumulated from metastatic seeding (green bar) until death. See Methods for more details. In this patient, the last identified subclones in the prostate were 5, 7, 8 and 9.

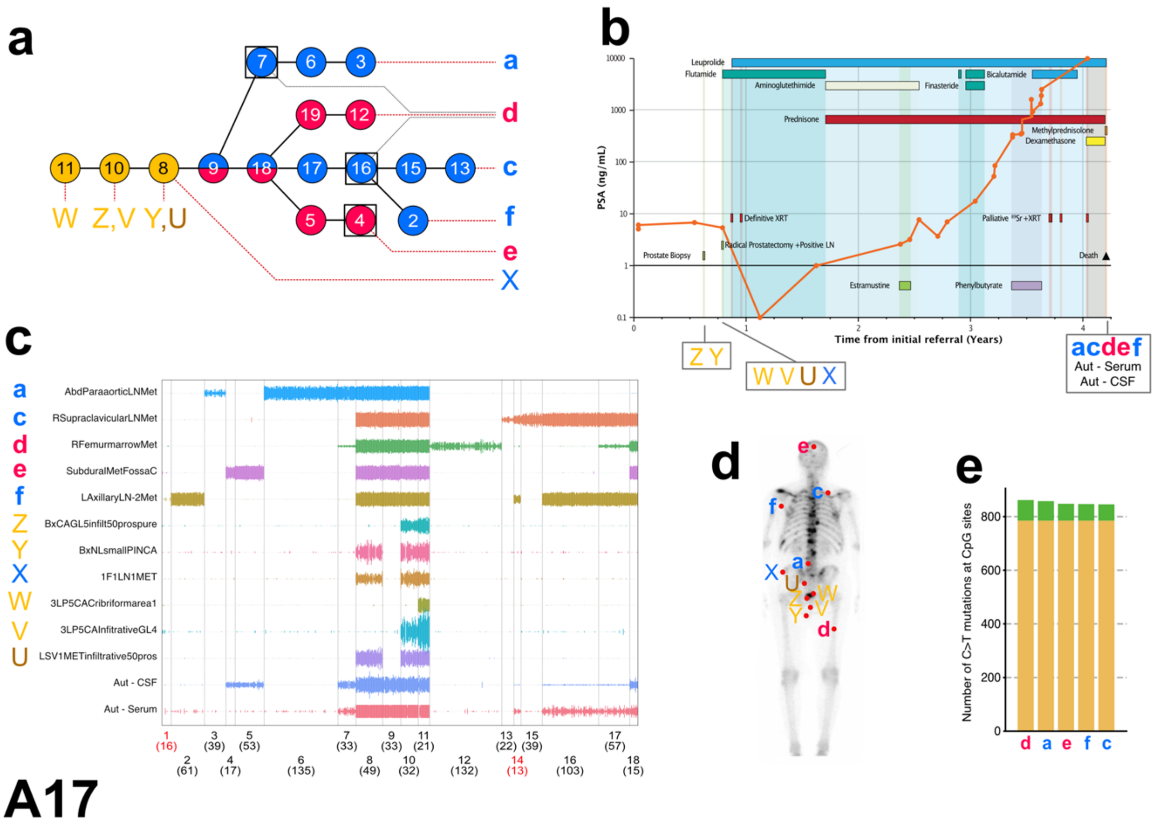


**Supplementary Figure 3 | Ancillary information for patient A17.** **a)** The phylogenetic tree (as in MP Fig. 1.) showing the subclonal cluster numbers identified with DPClust, displayed in subfigure **c**. Subclonal clusters are coloured as in MP Fig. 1. **b)** The clinical timeline from diagnosis to death showing PSA levels (red line, scale on y-axis) and treatments as annotated. The time of collection of samples is shown by the corresponding sample letter/name underneath the x-axis. **c)** The subclonal clusters identified by DPClust (x-axis), and the estimated cancer cell fraction (CCF) of each SNV (each individual bar) for the tumour in each of the samples (y-axis). Samples taken from body fluid were scaled so the CCF of the largest subclonal component was equal to 1, and the subfigure displays clipped values (at CCF=1) as SNV abundance displayed high variance. The numbers under the x-axis show the cluster number, and the number of SNV/indels assigned to the cluster in brackets. Numbers in red were not used to construct the phylogenetic tree as they contained >50% indels (see Methods). Original sample names are shown on the y-axis, along with sample letters used throughout this study. Body fluid samples denoted by time of sample - Aut: Autopsy; and body fluid type – CSF: Cerebrospinal fluid. See Supplementary Data 2 and Methods for more details on samples. **d)** Bone scan showing the approximate anatomical locations from which samples were taken, as indicated by their corresponding sample letter. **e)** Total number of C>T mutations in a CpG context observed in fresh frozen samples for patient A17, split into mutations accumulated from birth to first intra-prostatic branch or metastatic seeding event (yellow bar), and mutations accumulated from first branching event (grey bar) or metastatic seeding (green bar) until death. See Methods for more details. In this patient, subclone 8 was the last identified in the prostate.

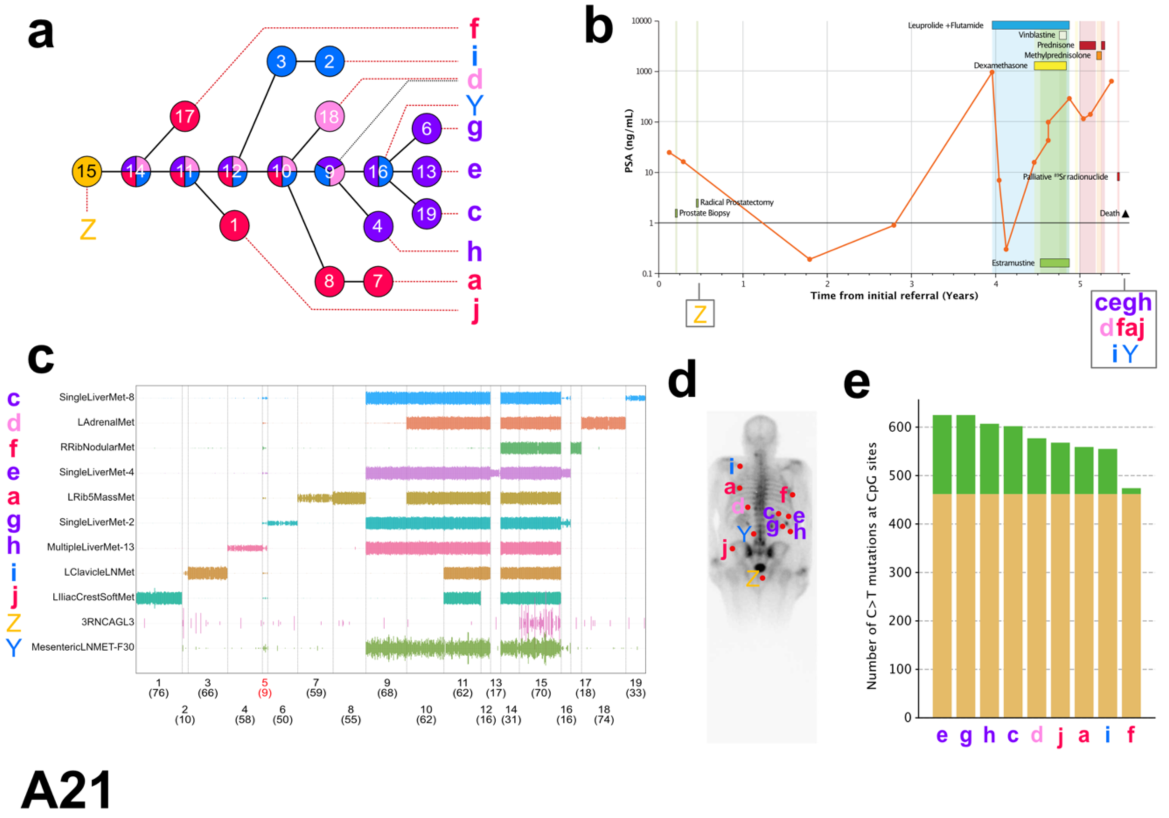

**Supplementary Figure 4 | Ancillary information for patient A21. a)** The phylogenetic tree (as in MP Fig. 1.) showing the subclonal cluster numbers identified with DPClust, displayed in subfigure **c**. Subclonal clusters are coloured as in MP Fig. 1. **b)** The clinical timeline from diagnosis to death showing PSA levels (red line, scale on y-axis) and treatments as annotated. The time of collection of samples is shown by the corresponding sample letter/name underneath the x-axis. **c)** The subclonal clusters identified by DPClust (x-axis), and the estimated cancer cell fraction (CCF) of each SNV (each individual bar) for the tumour in each of the samples (y-axis). Samples taken from body fluid were scaled so the CCF of the largest subclonal component was equal to 1, and the subfigure displays clipped values (at CCF=1) as SNV abundance displayed high variance. The numbers under the x-axis show the cluster number, and the number of SNV/indels assigned to the cluster in brackets. Numbers in red were not used to construct the phylogenetic tree as they contained >50% indels (see Methods). Original sample names are shown on the y-axis, along with sample letters used throughout this study. **d)** Bone scan showing the approximate anatomical locations from which samples were taken, as indicated by their corresponding sample letter. **e)** Total number of C>T mutations in a CpG context observed in fresh frozen samples for patient A21 split into mutations accumulated from birth to first intra-prostatic branch or metastatic seeding event (yellow bar), and mutations accumulated from first branching event (grey bar) or metastatic seeding (green bar) until death. See Methods for more details. In this patient, subclone 15 was the last identified in the prostate.

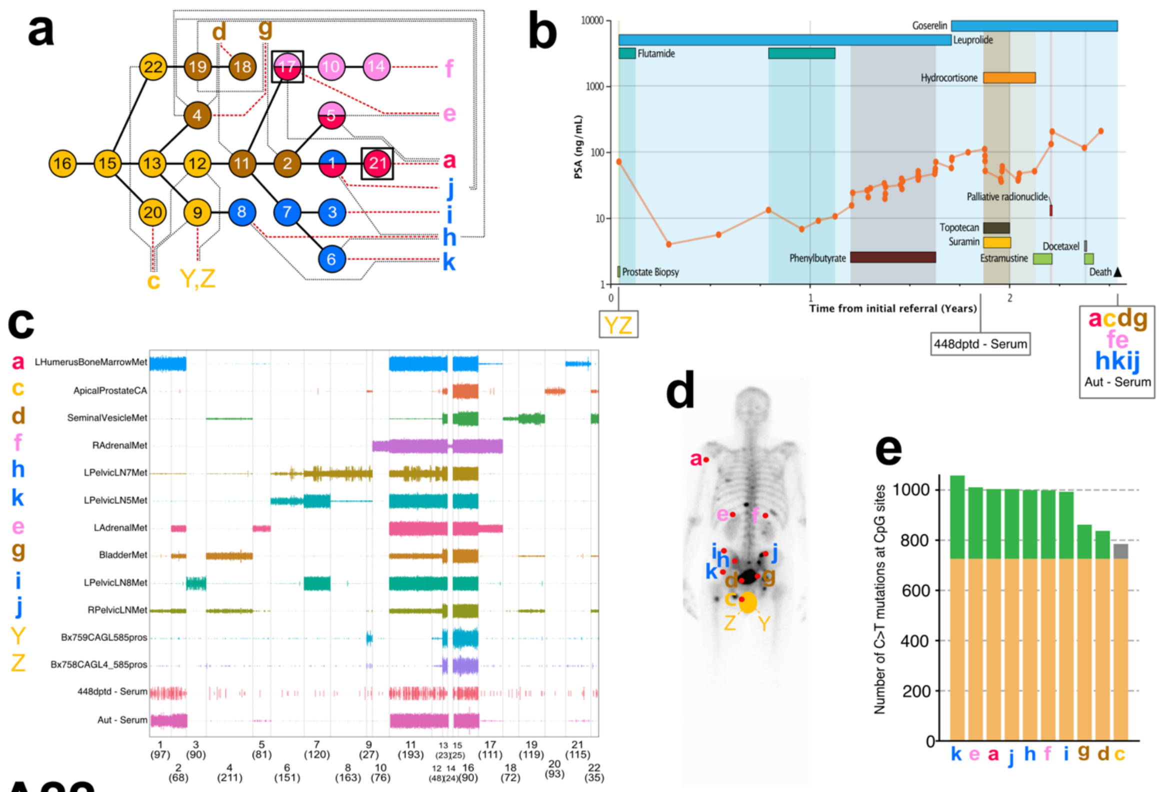

# A22

**Supplementary Figure 5 | Ancillary information for patient A22. a)** The phylogenetic tree (as in MP Fig. 1.) showing the subclonal cluster numbers identified with DPClust, displayed in subfigure **c**. Subclonal clusters are coloured as in MP Fig. 1. **b)** The clinical timeline from diagnosis to death showing PSA levels (red line, scale on y-axis) and treatments as annotated. The time of collection of samples is shown by the corresponding sample letter/name underneath the x-axis. **c)** The subclonal clusters identified by DPClust (x-axis), and the estimated cancer cell fraction (CCF) of each SNV (each individual bar) for the tumour in each of the samples (y-axis). Samples taken from body fluid were scaled so the CCF of the largest subclonal component was equal to 1, and the subfigure displays clipped values (at CCF=1) as SNV abundance displayed high variance. The numbers under the x-axis show the cluster number, and the number of SNV/indels assigned to the cluster in brackets. Numbers in red were not used to construct the phylogenetic tree as they contained >50% indels (see Methods). Original sample names are shown on the y-axis, along with sample letters used throughout this study. Body fluid samples denoted by time of sample. Body fluid samples denoted by time of sample - Aut: Autopsy; and body fluid type. See Supplementary Data 2 and Methods for more details on samples. **d)** Bone scan showing the approximate anatomical locations from which samples were taken, as indicated by their corresponding sample letter. **e)** Total number of C>T mutations in a CpG context observed in fresh frozen samples for patient A22, split into mutations accumulated from birth to first intra-prostatic branch or metastatic seeding event (yellow bar), and mutations accumulated from first branching event (grey bar) or metastatic seeding (green bar) until death. See Methods for more details. In this patient, extensive polyclonal seeding confounds the calculation of the SNVs observed at each branching event. As such, we identify that subclone 15 as the point where all metastatic branches diverge.

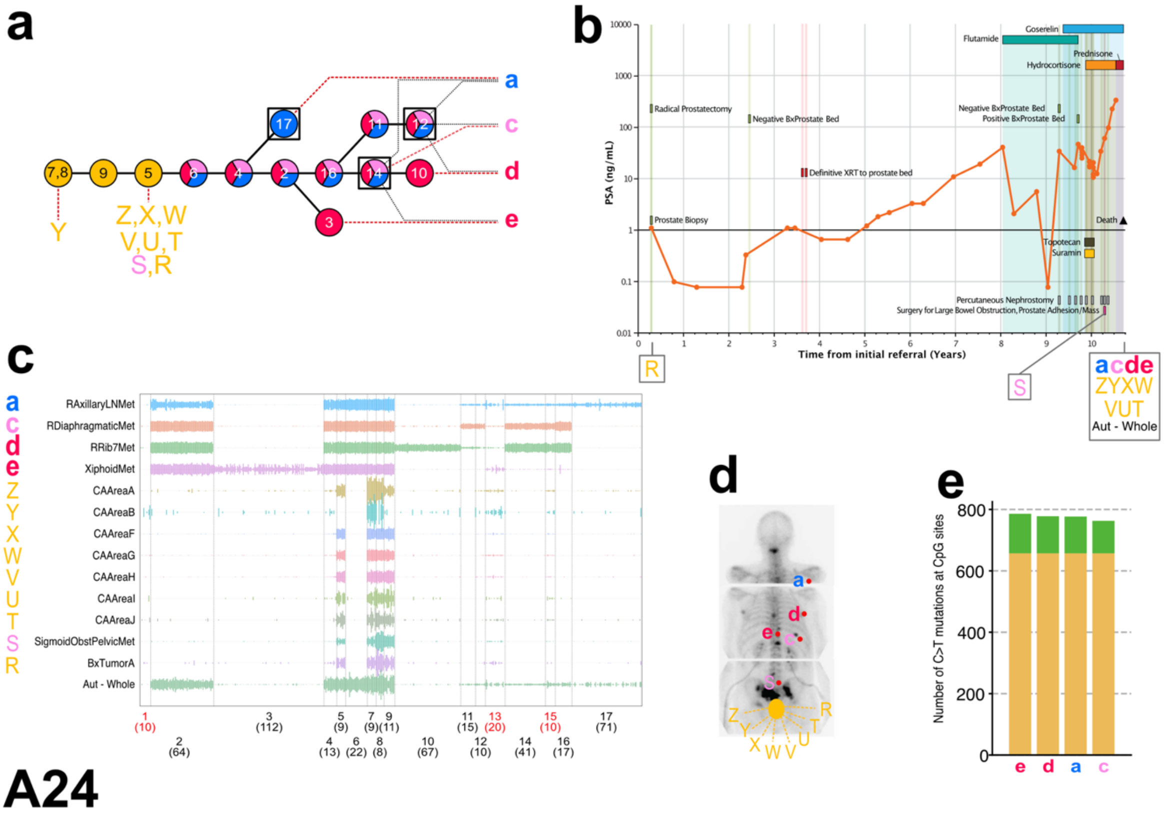

**Supplementary Figure 6 | Ancillary information for patient A24.** **a)** The phylogenetic tree (as in MP Fig. 1.) showing the subclonal cluster numbers identified with DPClust, displayed in subfigure **c**. Subclonal clusters are coloured as in MP Fig. 1. **b)** The clinical timeline from diagnosis to death showing PSA levels (red line, scale on y-axis) and treatments as annotated. The time of collection of samples is shown by the corresponding sample letter/name underneath the x-axis. **c)** The subclonal clusters identified by DPClust (x-axis), and the estimated cancer cell fraction (CCF) of each SNV (each individual bar) for the tumour in each of the samples (y-axis). Samples taken from body fluid were scaled so the CCF of the largest subclonal component was equal to 1, and the subfigure displays clipped values (at CCF=1) as SNV abundance displayed high variance. The numbers under the x-axis show the cluster number, and the number of SNV/indels assigned to the cluster in brackets. Numbers in red were not used to construct the phylogenetic tree as they contained >50% indels (see Methods). Original sample names are shown on the y-axis, along with sample letters used throughout this study. Body fluid samples denoted by time of sample - Aut: Autopsy; and body fluid type - Whole: Whole Blood. See Supplementary Data 2 and Methods for more details on samples. **d)** Bone scan showing the approximate anatomical locations from which samples were taken, as indicated by their corresponding sample letter. **e)** Total number of C>T mutations in a CpG context observed in fresh frozen samples for patient A24, split into mutations accumulated from birth to first intra-prostatic branch or metastatic seeding event (yellow bar), and mutations accumulated from first branching event (grey bar) or metastatic seeding (green bar) until death. See Methods for more details. In this patient, subclone 5 was the last identified in the prostate.

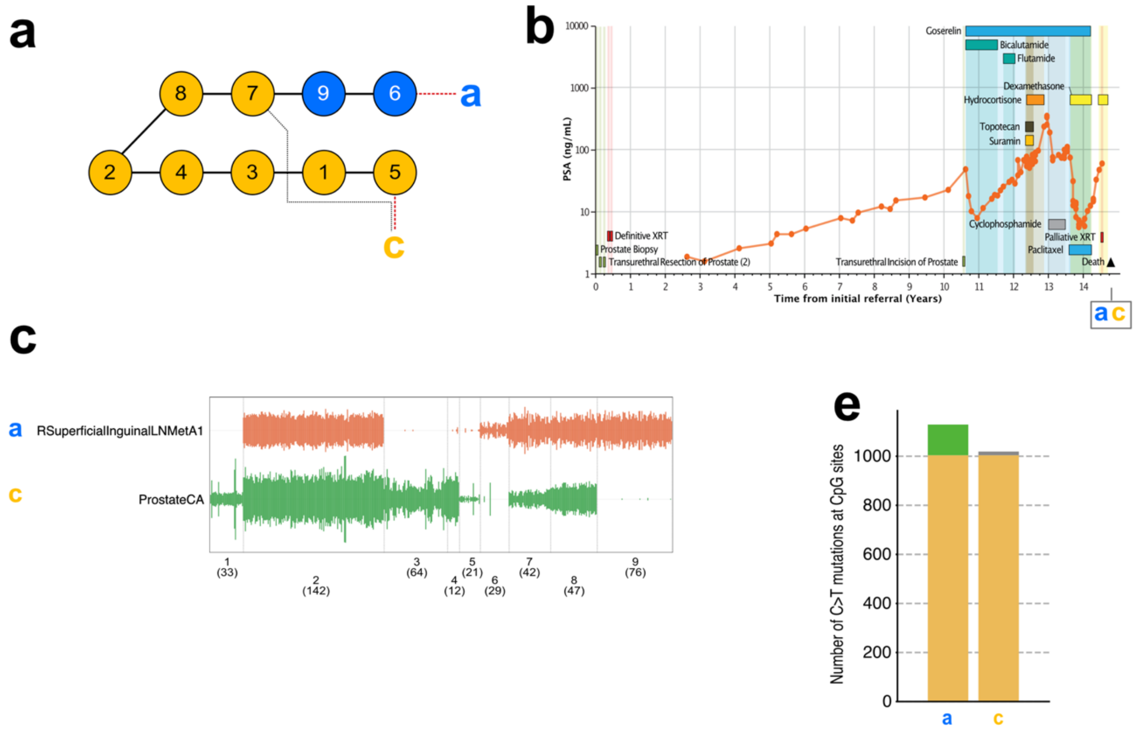


# A29

**Supplementary Figure 7 | Ancillary information for patient A29. a)** The phylogenetic tree (as in MP Fig. 1.) showing the subclonal cluster numbers identified with DPClust, displayed in subfigure **c**. Subclonal clusters are coloured as in MP Fig. 1. **b)** The clinical timeline from diagnosis to death showing PSA levels (red line, scale on y-axis) and treatments as annotated. The time of collection of samples is shown by the corresponding sample letter/name underneath the x-axis. **c)** The subclonal clusters identified by DPClust (x-axis), and the estimated cancer cell fraction (CCF) of each SNV (each individual bar) for the tumour in each of the samples (y-axis). Samples taken from body fluid were scaled so the CCF of the largest subclonal component was equal to 1, and the subfigure displays clipped values (at CCF=1) as SNV abundance displayed high variance. The numbers under the x-axis show the cluster number, and the number of SNV/indels assigned to the cluster in brackets. Numbers in red were not used to construct the phylogenetic tree as they contained >50% indels (see Methods). Original sample names are shown on the y-axis, along with sample letters used throughout this study. Body fluid samples denoted by time of sample - Aut: Autopsy; and body fluid type – CSF: Cerebrospinal fluid. See Supplementary Data 2 and Methods for more details on samples. **d)** is not present for this patient due to the lack of a bone scan. **e)** Total number of C>T mutations in a CpG context observed in fresh frozen samples for patient A29, split into mutations accumulated from birth to first intra-prostatic branch or metastatic seeding event (yellow bar), and mutations accumulated from first branching event (grey bar) or metastatic seeding (green bar) until death. See Methods for more details. In this patient, the intra-prostatic branch that leads to both the intra-prostatic and metastatic lineages occurred at subclone 2.

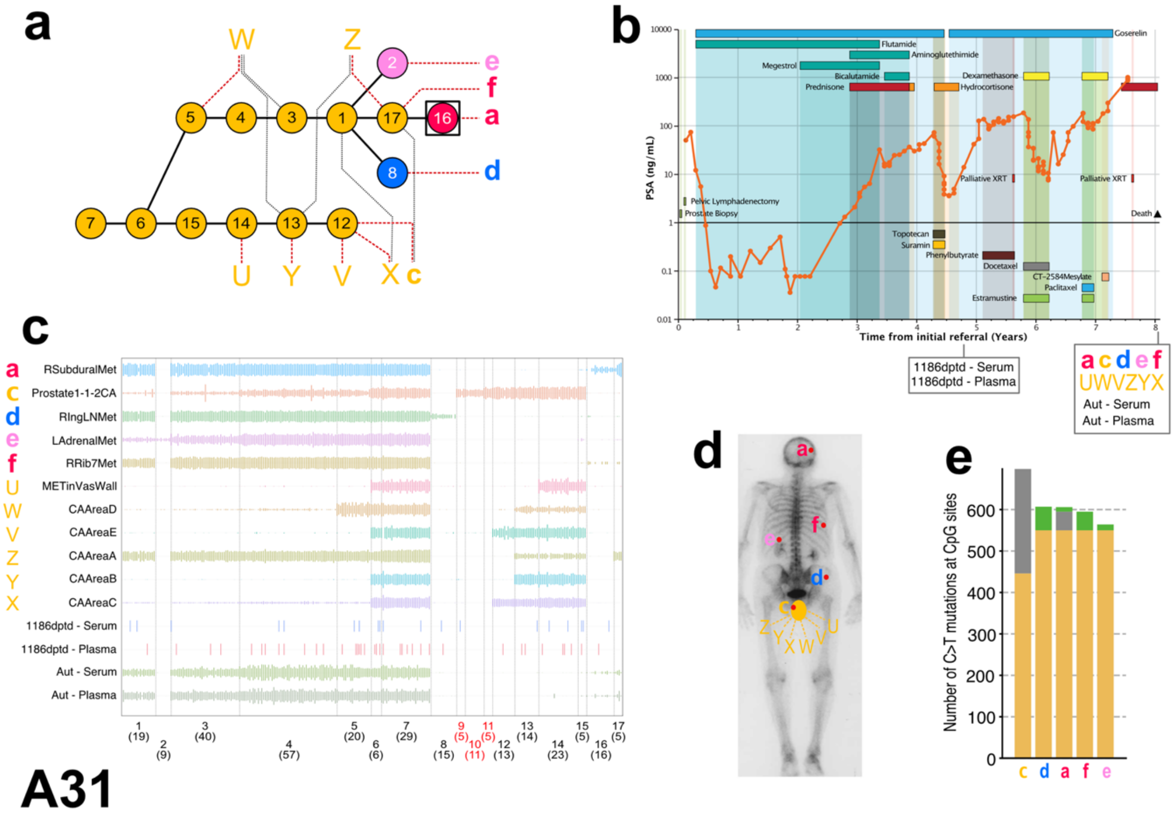


**Supplementary Figure 8 | Ancillary information for patient A31. a)** The phylogenetic tree (as in MP Fig. 1.) showing the subclonal cluster numbers identified with DPClust, displayed in subfigure **c**. Subclonal clusters are coloured as in MP Fig. 1. **b)** The clinical timeline from diagnosis to death showing PSA levels (red line, scale on y-axis) and treatments as annotated. The time of collection of samples is shown by the corresponding sample letter/name underneath the x-axis. **c)** The subclonal clusters identified by DPClust (x-axis), and the estimated cancer cell fraction (CCF) of each SNV (each individual bar) for the tumour in each of the samples (y-axis). Samples taken from body fluid were scaled so the CCF of the largest subclonal component was equal to 1, and the subfigure displays clipped values (at CCF=1) as SNV abundance displayed high variance. The numbers under the x-axis show the cluster number, and the number of SNV/indels assigned to the cluster in brackets. Numbers in red were not used to construct the phylogenetic tree as they contained >50% indels (see Methods). Original sample names are shown on the y-axis, along with sample letters used throughout this study. Body fluid samples denoted by time of sample - Aut: Autopsy, dptd: days prior to death; and body fluid type. See Supplementary Data 2 and Methods for more details on samples. **d)** Bone scan showing the approximate anatomical locations from which samples were taken, as indicated by their corresponding sample letter. **e)** Total number of C>T mutations in a CpG context observed in fresh frozen samples for patient A31, split into mutations accumulated from birth to first intra-prostatic branch or metastatic seeding event (yellow bar), and mutations accumulated from first branching event (grey bar) or metastatic seeding (green bar) until death. See Methods for more details. In this patient, an intra-prostatic branch occurs at subclone 6. Metastatic seeding was first observed at subclone 1 and again at subclone 17.

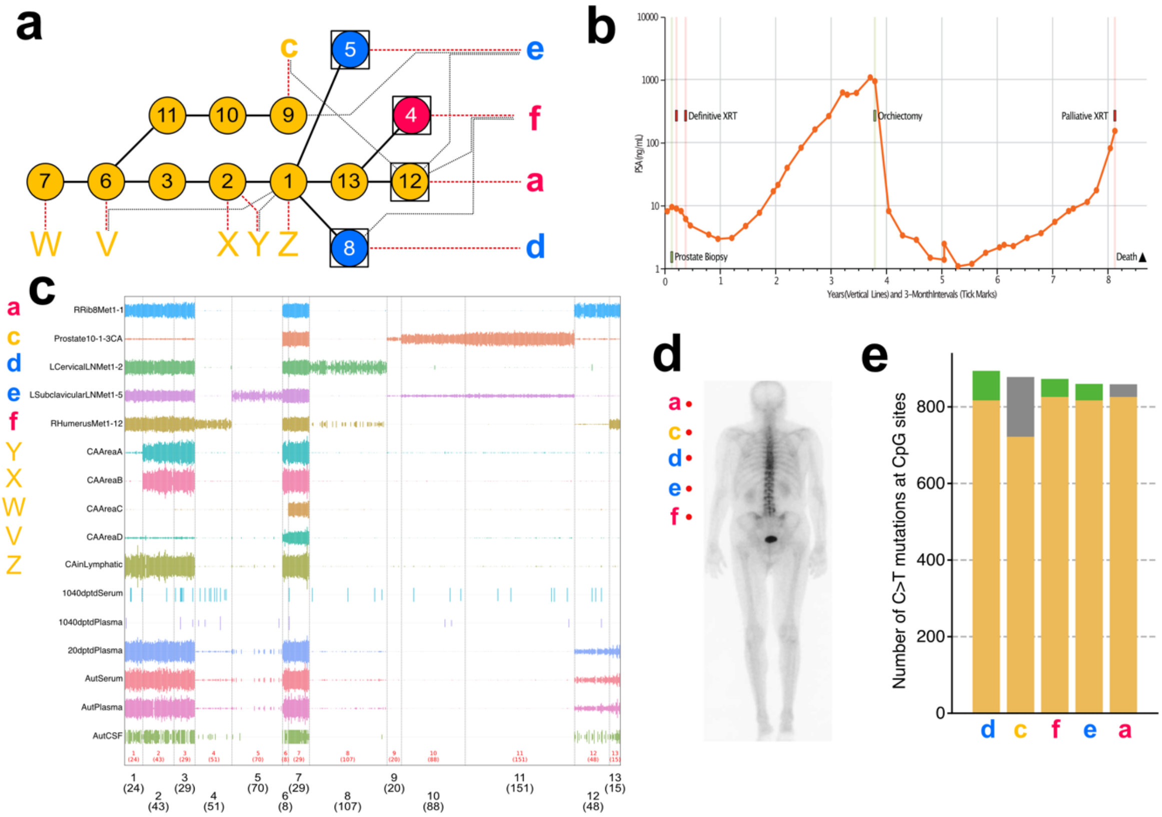


**Supplementary Figure 9 | Ancillary information for patient A32.** **a)** The phylogenetic tree (as in MP Fig. 1.) showing the subclonal cluster numbers identified with DPClust, displayed in subfigure **c**. Subclonal clusters are coloured as in MP Fig. 1. **b)** The clinical timeline from diagnosis to death showing PSA levels (red line, scale on y-axis) and treatments as annotated. The time of collection of samples is shown by the corresponding sample letter/name underneath the x-axis. **c)** The subclonal clusters identified by DPClust (x-axis), and the estimated cancer cell fraction (CCF) of each SNV (each individual bar) for the tumour in each of the samples (y-axis). Samples taken from body fluid were scaled so the CCF of the largest subclonal component was equal to 1, and the subfigure displays clipped values (at CCF=1) as SNV abundance displayed high variance. The numbers under the x-axis show the cluster number, and the number of SNV/indels assigned to the cluster in brackets. Numbers in red were not used to construct the phylogenetic tree as they contained >50% indels (see Methods). Original sample names are shown on the y-axis, along with sample letters used throughout this study. Body fluid samples denoted by time of sample - Aut: Autopsy, dptd: days prior to death; and body fluid type – CSF: Cerebrospinal fluid. See Supplementary Data 2 and Methods for more details on samples. **d)** Bone scan showing the approximate anatomical locations from which samples were taken, as indicated by their corresponding sample letter. **e)** Total number of C>T mutations in a CpG context observed in fresh frozen samples for patient A32, split into mutations accumulated from birth to first intra-prostatic branch or metastatic seeding event (yellow bar), and mutations accumulated from first branching event (grey bar) or metastatic seeding (green bar) until death. See Methods for more details. In this patient, the intra-prostatic branch that leads to the lineage ending at subclone 9 occurred at subclone 6. Metastatic seeding was first observed at subclone 1 and again at subclones 13 and 12.

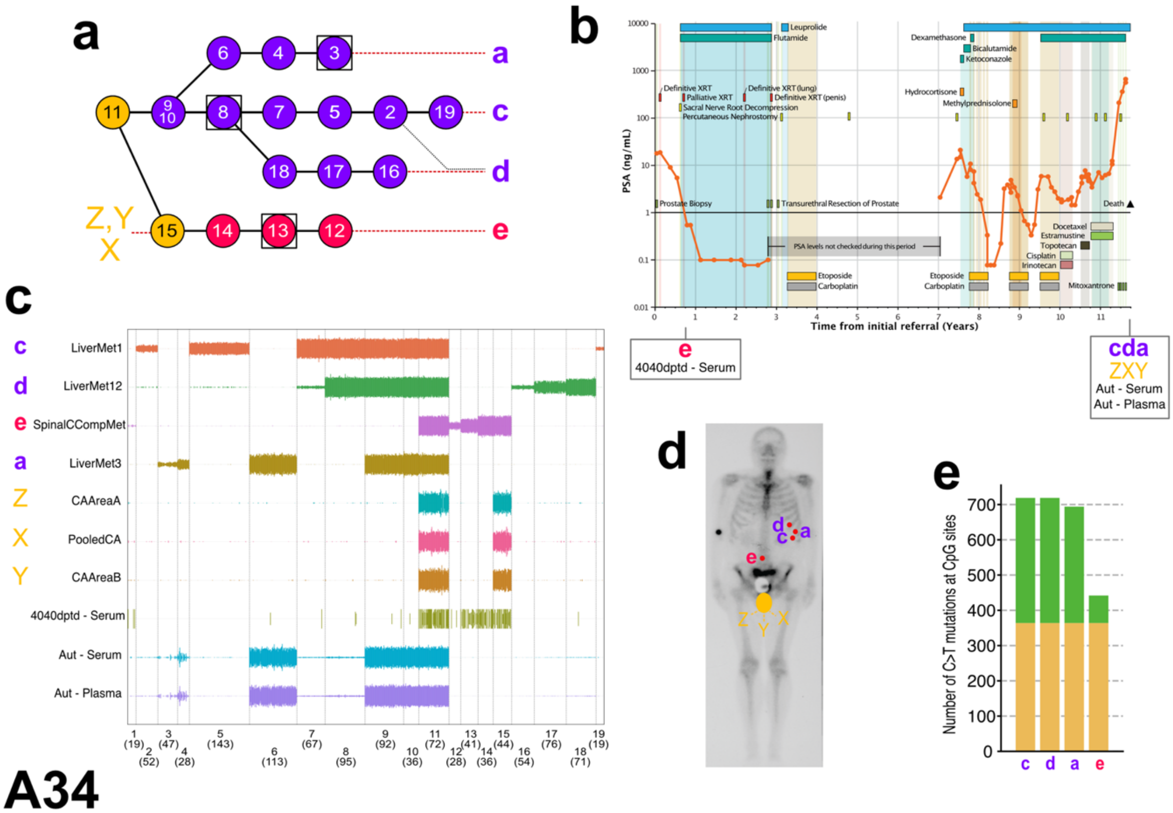


**Supplementary Figure 10 | Ancillary information for patient A34.** **a)** The phylogenetic tree (as in MP Fig. 1.) showing the subclonal cluster numbers identified with DPClust, displayed in subfigure **c**. Subclonal clusters are coloured as in MP Fig. 1. **b)** The clinical timeline from diagnosis to death showing PSA levels (red line, scale on y-axis) and treatments as annotated. The time of collection of samples is shown by the corresponding sample letter/name underneath the x-axis. **c)** The subclonal clusters identified by DPClust (x-axis), and the estimated cancer cell fraction (CCF) of each SNV (each individual bar) for the tumour in each of the samples (y-axis). Samples taken from body fluid were scaled so the CCF of the largest subclonal component was equal to 1, and the subfigure displays clipped values (at CCF=1) as SNV abundance displayed high variance. The numbers under the x-axis show the cluster number, and the number of SNV/indels assigned to the cluster in brackets. Numbers in red were not used to construct the phylogenetic tree as they contained >50% indels (see Methods). Original sample names are shown on the y-axis, along with sample letters used throughout this study. Body fluid samples denoted by time of sample - Aut: Autopsy, dptd: days prior to death; and body fluid type. See Supplementary Data 2 and Methods for more details on samples. **d)** Bone scan showing the approximate anatomical locations from which samples were taken, as indicated by their corresponding sample letter. **e)** Total number of C>T mutations in a CpG context observed in fresh frozen samples for patient A34, split into mutations accumulated from birth to first intra-prostatic branch or metastatic seeding event (yellow bar), and mutations accumulated from first branching event (grey bar) or metastatic seeding (green bar) until death. See Methods for more details. In this patient, the intra-prostatic branch that leads the lineage ending at subclone 12 occurred at subclone 11. This lineage was eradicated by treatment. Subclone 11 also corresponded to the last observed set of intra-prostatic SNVs observed in the liver metastases present at time of death.

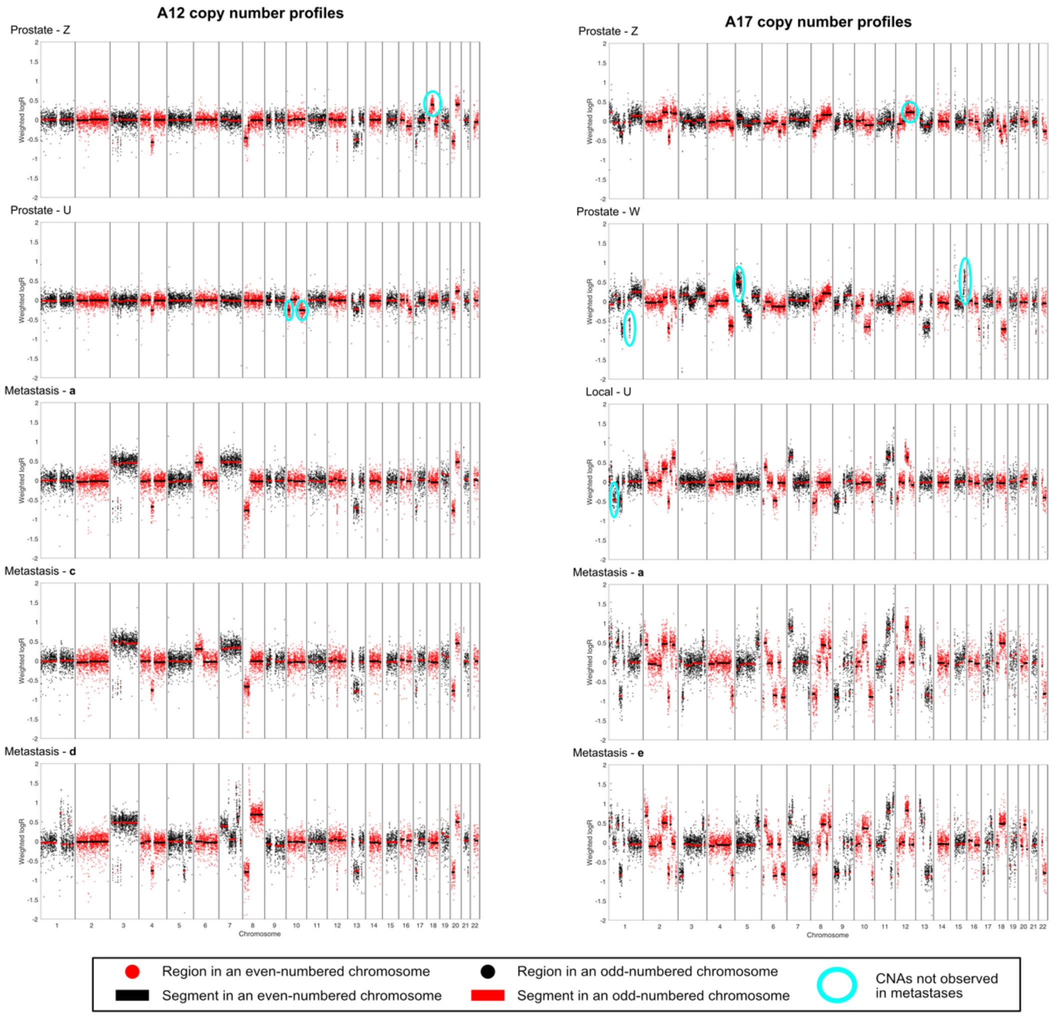


**Supplementary Figure 11 | Copy number profiles of samples from A12 and A17.** Each plot shows the weighted logR values of windowed reads in the targeted regions and corresponding segments for A12 (left) and A17 (right), calculated by CNVKit (details in Methods). CNAs observed in intra-prostatic samples are circled, and provide evidence that tumour in these samples diverged from the metastatic lineage within the prostate.

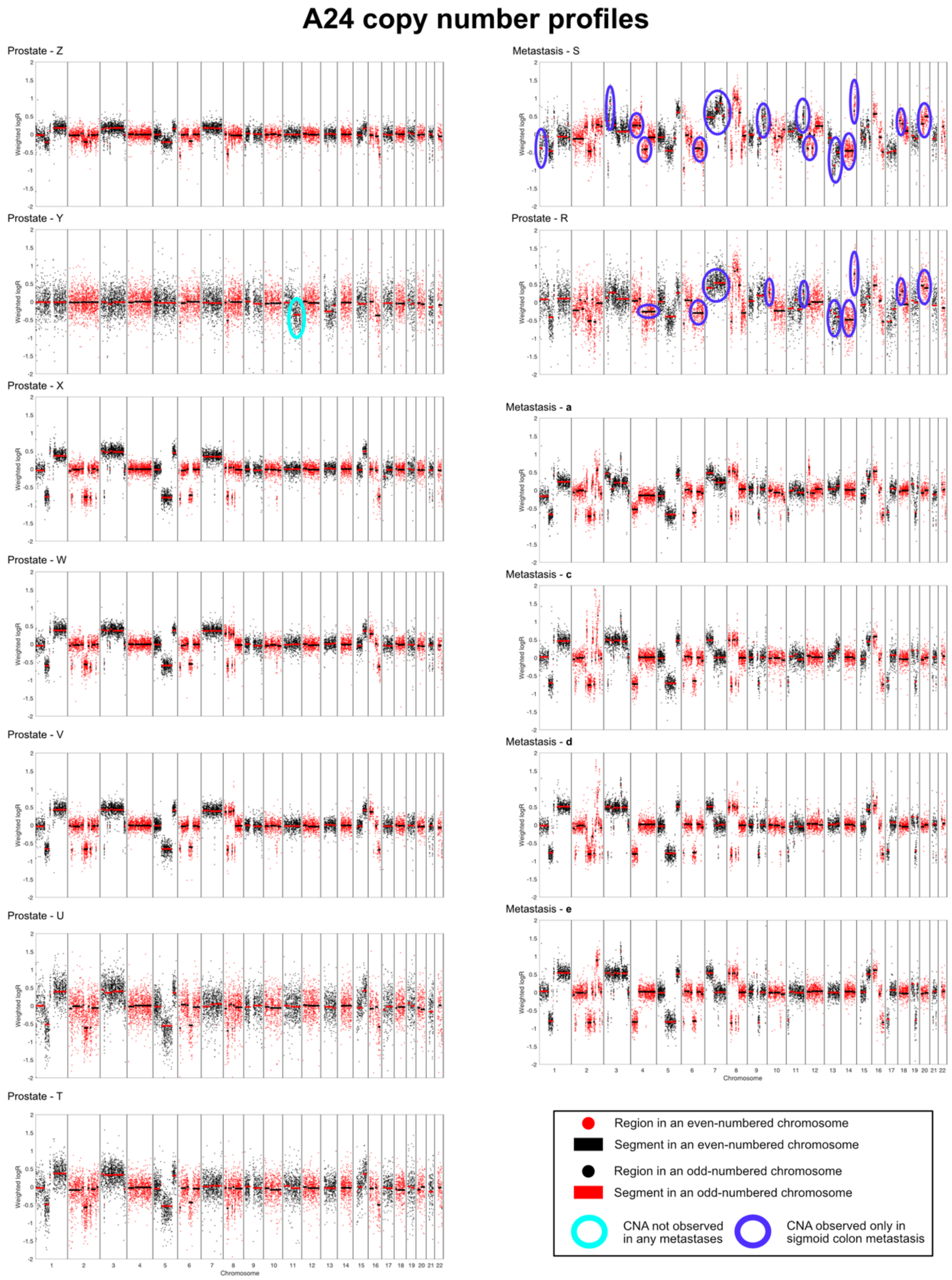


**Supplementary Figure 12 | Copy number profiles of samples from A24.** Each plot shows the weighted logR values of windowed reads in the targeted regions and corresponding segments for A24, calculated by CNVKit (details in Methods). A CNA observed in intra-prostatic samples are circled in cyan, and provide evidence that a tumour in these samples diverged from the metastatic lineage within the prostate. CNAs encircled in violet are those observed in the sigmoid colon metastasis S. This provides evidence that this sample, and prostate biopsy sample R, are on a divergent lineage from the rest of the sampled metastases.

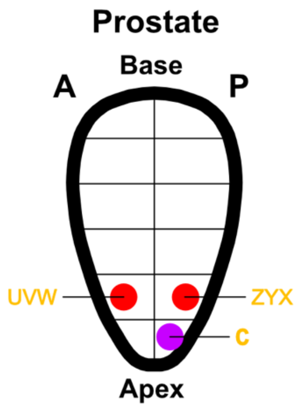


**Supplementary Figure 13 | Schematic for prostatic locations of samples in A31.** Due to the small size, the prostate of A31 was divided into five sections. All samples were taken from the lower two sections, with approximate locations of the paraffin embedded samples indicated by the red circle and fresh frozen samples by the purple circle. Sample letters taken from these regions are indicated in the diagram.

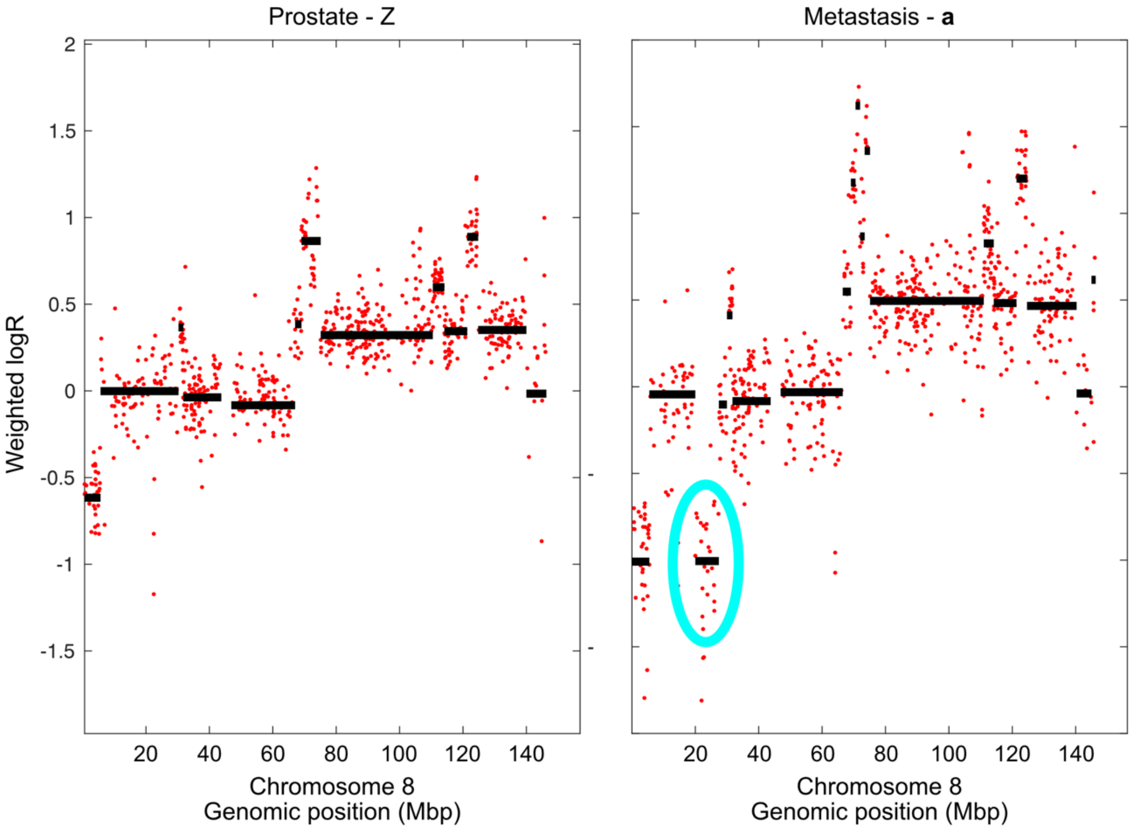


**Supplementary Figure 14 | Copy number profiles of chromosome 8 for samples a and Z in A10.** Each plot shows the weighted logR values of windowed reads in the targeted regions and corresponding segments for chromosome 8 in patient A10, calculated by CNVKit (details in Methods). An additional CNA covering the *NKX3-1* locus in sample **a** is circled in cyan.

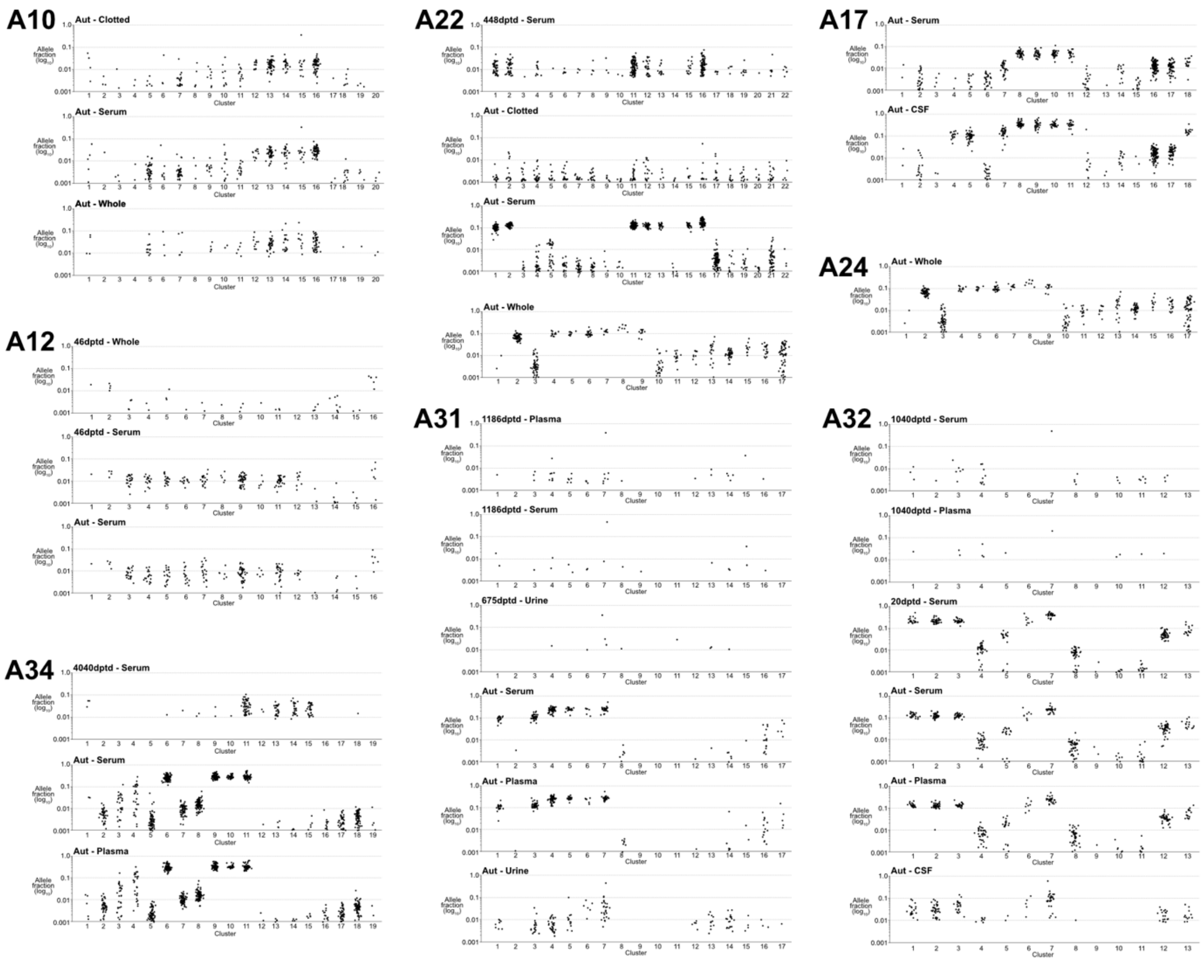


**Supplementary Figure 15 | Variant allele fractions for body fluids across 8 patients.** Each individual plot corresponds to single body fluid sample as denoted by the time the sample was taken (Aut: Autopsy, *dptd: * days prior to death) and the body fluid type (Urine, Plasma, Serum, Whole: Whole Blood, Clotted: Clotted Blood, CSF: Cerebrospinal Fluid). The patient number is shown at the start of a block of plots. Each point shows the variant allele fraction of a single variant base on the target panel which displayed allelic fraction > 0.
