# Supplementary material for: Prostate cancer evolution from multilineage primary to single lineage metastases with implications for liquid biopsy
Source: Nat Commun. 2020 Oct 8;11:5070. doi: 10.1038/s41467-020-18843-5 (PMC7545111; doi:10.1038/s41467-020-18843-5)
Supplement: Supplementary file 2 — Description of Additional Supplementary Files [file 41467_2020_18843_MOESM2_ESM.pdf]

Description of Additional Supplementary Files

Title: Supplementary Data 1

Description: Estimated CCFs for each subclone in each sample for each patient. CCFs were estimated using multi-sample DPClust for all tissue samples in each individual patient. These CCF values were used to construct the phylogenetic trees used throughout the study.

Title: Supplementary Data 2

Description: Sample Summary. Excel sheet containing a list of all samples and sample processing data. The header row defines what is contained in each column.

Title: Supplementary Data 3

Description: Images and summary data of regions of interest (ROI) laser-dissected from paraffinembedded tissues for the study.

Title: Supplementary Data 4

Description: List of genomic regions targeted for deep sequencing with genome position references to GRCh38.

Title: Supplementary Data 5

Description: List of coding indels targeted with genome position references to GRCh38.

Title: Supplementary Data 6

Description: List of coding substitions targeted with genome references to GRCh38.

Title: Supplementary Data 7

Description: Annotated Dirichlet process cluster figures for all samples for each subject, including EGA A and EGA B samples. The height of each bar denotes the relative CCFs of an individual SNV or indel.
