# Supplementary material for: Prostate cancer evolution from multilineage primary to single lineage metastases with implications for liquid biopsy
Source: Nat Commun. 2020 Oct 8;11:5070. doi: 10.1038/s41467-020-18843-5 (PMC7545111; doi:10.1038/s41467-020-18843-5)
Supplement: Supplementary file 10 — Reporting Summary [file 41467_2020_18843_MOESM10_ESM.pdf]

natureresearch

Corresponding author(s): David C Wedge, G Steven Bova

Last updated by author(s): 13/8/20

Reporting Summary

Nature Research wishes to improve the reproducibility of the work that we publish. This form provides structure for consistency and transparency in reporting. For further information on Nature Research policies, seeAuthors & Referees and theEditorial Policy Checklist .

Statistics

For all statistical analyses, confirm that the following items are present in the figure legend, table legend, main text, or Methods section.

n/a	Confirmed	
x	☐	The exact sample size (*n*) for each experimental group/condition, given as a discrete number and unit of measurement
x	☐	A statement on whether measurements were taken from distinct samples or whether the same sample was measured repeatedly
x	☐	The statistical test(s) used AND whether they are one- or two-sided<br>*Only common tests should be described solely by name; describe more complex techniques in the Methods section.*
x	☐	A description of all covariates tested
x	☐	A description of any assumptions or corrections, such as tests of normality and adjustment for multiple comparisons
x	☐	A full description of the statistical parameters including central tendency (e.g. means) or other basic estimates (e.g. regression coefficient) AND variation (e.g. standard deviation) or associated estimates of uncertainty (e.g. confidence intervals)
x	☐	For null hypothesis testing, the test statistic (e.g. *F*, *t*, *r*) with confidence intervals, effect sizes, degrees of freedom and *P* value noted<br>*Give P values as exact values whenever suitable.*
x	☐	For Bayesian analysis, information on the choice of priors and Markov chain Monte Carlo settings
x	☐	For hierarchical and complex designs, identification of the appropriate level for tests and full reporting of outcomes
x	☐	Estimates of effect sizes (e.g. Cohen's *d*, Pearson's *r*), indicating how they were calculated

Our web collection on statistics for biologists contains articles on many of the points above.

Software and code

Policy information about availability of computer code

Data collection

DNA preprocessing and alignment performed using:
Java OpenJDK Runtime Environment (build 1.8.0_65-b17)
Python 2.7.5
FastQC v0.11.5
Trimmomatic trimmomatic-0.32
BWA-MEME 0.7.12-r1039
GATK GATK-3.5-1
PICARD 1.63(1131)
bamUtil Version: 1.0.14
GRCh37_to_GRCh38.chain Download date 18/12/2015
CrossMap.py CrossMap v0.1.8
samtools 1.3.1 (using htslib 1.3.1)
bam-readcount 0.8.0-unstable-5-1b9c52c-dirty (commit 1b9c52c-dirty)

Identification of C>T mutations in a CpG context:
BSgenome (1.48.0)
Biostrings (v2.48.0)
R (v3.5.1)

Data analysis

Battenberg algorithm used is available at https://github.com/cancerit/cgpBattenberg
DPClust used is available at https://github.com/Wedge-Oxford/dpclust
CNVKit (v0.9.1)
R (v3.5.1)

For manuscripts utilizing custom algorithms or software that are central to the research but not yet described in published literature, software must be made available to editors/reviewers. We strongly encourage code deposition in a community repository (e.g. GitHub). See the Nature Research guidelines for submitting code & software for further information.

Data

Policy information about availability of data

All manuscripts must include a data availability statement. This statement should provide the following information, where applicable:

- Accession codes, unique identifiers, or web links for publicly available datasets
- A list of figures that have associated raw data
- A description of any restrictions on data availability

Targeted sequencing data created for this study has been deposited to European Genome Archive (EGA) as EGAD00001005381 and EGAD00001005382 (TenMenDeep EGA Datasets A and B respectively) and is publicly available.
Gene names taken from UCSC Genome Browser, Cancer Genetics Web, chromosome 8, GRCh38
Whole genome sequence data is an external data set located in the (EGA) under accession code EGAD00001000891. This falls under remit of the ICGC Data Access Compliance Office and requires registration (details available on EGA under this accession code).

Field-specific reporting

Please select the one below that is the best fit for your research. If you are not sure, read the appropriate sections before making your selection.

☒ Life sciences ☐ Behavioural & social sciences ☐ Ecological, evolutionary & environmental sciences

For a reference copy of the document with all sections, see nature.com/documents/nr-reporting-summary-flat.pdf

Life sciences study design

All studies must disclose on these points even when the disclosure is negative.

Sample size	163 tissue and body fluid samples were taken for use in this study. The PELICAN integrated clinical-molecular autopsy study of lethal prostate cancer is a natural history study. Selection of the types and numbers of samples included in this manuscript was aimed at obtaining representation of cancer cell behavior over time and anatomy sufficient for advancing understanding of prostate cancer evolution. All available body fluids from the ten men were included, and availability of samples was dependent on ad hoc patient clinic visits and PELICAN clinical coordinator availability. Autopsy samples selected for study were based on studying as complete a range of primary, metastatic, and noncancerous samples as possible, within the constraints of the budget. As this is a descriptive study and no hypotheses will be tested, a sample size calculation is not applicable.
Data exclusions	Samples were excluded from the analysis using pre-established critera:<br>a) unable to be used for DNA sequencing<br>b) contamination by extraneous human DNA<br>In total, 13 samples failed library preparation and so were excluded under criteria a) and we identified in one sample that were not found in other samples from that patient, indicating possible contamination from an external source and so was excluded under criteria b)
Replication	Samples were taken from different anatomical sites or sampled at different times (body fluids) and so direct biological replication with exactly the same conditions was not possible. Multiple DNA sequencing of the same sample is not possible as the procedure destroys the sample and no technical replication was possible. Single nucleotide variants were only included in the analysis if they were identified in 2 or more samples from the same patient as it is highly unlikely that identification of the same base change at the same genomic position in two separate samples would arise by error.
Randomization	No groups were allocated and no statistical analysis performed so randomisation was not necessary
Blinding	No groups were allocated and no statistical analysis performed so blinding was not necessary

Reporting for specific materials, systems and methods

We require information from authors about some types of materials, experimental systems and methods used in many studies. Here, indicate whether each material, system or method listed is relevant to your study. If you are not sure if a list item applies to your research, read the appropriate section before selecting a response.

Materials & experimental systems

n/a	Involved in the study
☒	☐ Antibodies
☒	☐ Eukaryotic cell lines
☒	☐ Palaeontology
☒	☐ Animals and other organisms
☐	☒ Human research participants
☒	☐ Clinical data

Methods

n/a	Involved in the study
☒	☐ ChIP-seq
☒	☐ Flow cytometry
☒	☐ MRI-based neuroimaging

Human research participants

Policy information about studies involving human research participants

Population characteristics	10 men from the PELICAN (Project to ELIminate lethal CANcer) integrated clinical-molecular autopsy study of metastatic prostate cancer were the subjects of the current study. The mean age of the study subjects at the time of diagnosis of prostate cancer was 61 years. 1 subject reported White Hispanic ancestry, 2 reported Black non-Hispanic ancestry, and 7 reported White non-Hispanic ancestry. 1 subject was treated in a community practice setting only, 4 were treated primarily in an oncology specialty clinic, and 5 were treated primarily in a community setting with consultation with an oncology specialty clinic. A17 had a germline BRCA1 mutation.
Recruitment	Candidate study patients were identified by clinicians in three care settings (oncology specialty clinic, community setting, or community setting with consultation with an oncology specialty clinic). No clear biases in presentation of the study, or consent to participate in the study were evident.
Ethics oversight	John Hopkins Medicine IRB

Note that full information on the approval of the study protocol must also be provided in the manuscript.
